# A pathogenic variant of *AMOT* leads to isolated X-linked congenital hydrocephalus due to N-terminal truncation

**DOI:** 10.1172/JCI179438

**Published:** 2025-09-02

**Authors:** Nurcan Hastar, Hagit Daum, Nikoletta Kardos-Török, Gael Ganz, Leon Obendorf, Peter Vajkoczy, Orly Elpeleg, Petra Knaus

**Affiliations:** 1 Institute for Chemistry and Biochemistry, Freie Universitaet Berlin, Berlin, Germany.; 2 Department of Genetics, Hadassah Hebrew Medical Center and the Faculty of Medicine, Jerusalem, Israel.; 3 Department of Neurosurgery with Pediatric Neurosurgery, Charité Universitätsmedizin, Berlin, Germany.

**Keywords:** Cell biology, Genetics, Genetic diseases, Molecular diagnosis, Tight junctions

## Abstract

Congenital hydrocephalus is a life-threatening condition that might affect brain development by increasing the pressure on the brain parenchyma. Here, we describe 6 male patients from 1 family, all presenting with an isolated X-linked congenital hydrocephalus. Exome sequencing identified a likely pathogenic variant of angiomotin (*AMOT*) that segregated with the phenotype in the extended family. We show that the variant, affecting the first methionine, translated into a shorter AMOT protein lacking 91 amino acids from the N-terminus. Mechanistically, we unraveled that the absence of the N-terminus leads to abnormally increased AMOT protein levels due to the loss of both the N-degron degradation signal and the tankyrase-binding domain. Altered degradation of AMOT disrupted the barrier integrity of the cells. Thus, the identified *AMOT* variant likely underlies the clinical presentation of isolated X-linked hydrocephalus in this family, and our data underscore the importance of tight regulation of AMOT protein level in the brain. AMOT now joins the list of genes involved in congenital hydrocephalus in humans. These findings are instrumental for the genetic counseling of affected families.

## Abstract 2

We identified a genetic mutation in the AMOT gene linked to inherited hydrocephalus, revealing how disrupted protein regulation may impair brain development and barrier function.

## Graphical abstract

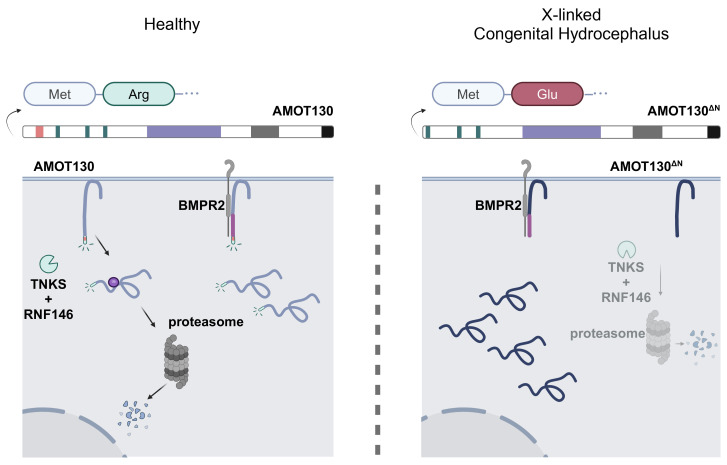

## Introduction

Hydrocephalus is a neurological disorder manifested by cerebral ventricular expansion and caused by impaired cerebrospinal fluid (CSF) circulation or absorption. The prevalence of congenital hydrocephalus is 1–32 in 10,000 births and increases to 1.1 in 1,000 when including infants diagnosed up to the age of 1 year, excluding neural tube defects and extrinsic factors (1). Acquired hydrocephalus caused by factors such as hemorrhage, infection, or neoplasia accounts for 50% of the reported cases, and the other half are termed “intrinsic” and thought to be caused by abnormal prenatal development. Hydrocephalus can be communicating or obstructive (i.e., noncommunicating); that latter is more common. More detailed and explanatory categorizations are used, based on the age of onset and the levels of CSF pressure, as expressed by ventricular width (2).

Of the types of congenital hydrocephalus, 75% of cases are syndromic. Of these, 30% are caused by chromosomal anomalies and 25% are nonsyndromic. In 40% of the nonsyndromic cases, isolated hydrocephalus is thought to have a genetic cause (3); however, the number of identified genes underlying this condition is relatively small. Pathogenic variants in *L1CAM* account for 5% to 10% of nonsyndromic X-linked hydrocephalus in male infants (4). More than 300 pathogenic variants in *L1CAM* account for a wide spectrum of hydrocephalus phenotypes with variable degrees and positions of the obstruction (5, 6). Of note, all phenotypes include developmental delay and intellectual disability.

Isolated hydrocephalus can also be caused by biallelic pathogenic variants of the *MPDZ* gene, heretofore reported in 5 consanguineous families with isolated hydrocephalus inherited in an autosomal recessive manner (3). The *MPDZ* variants caused ependymal malformations along the aqueduct of Sylvius, leading to hydrocephalus. In all cases, diagnosis was made prenatally, and the affected fetuses were terminated, thus no developmental data are available.

MPDZ, also known as MUPP1, is an important tight junction protein expressed strongly in the choroid plexus and embryonic brain (7). Tight junction proteins are essential in holding cellular alignment firm via cell-to-cell adhesion of PDZ proteins such as MPDZ, thus preventing the diffusion of solutes, lipids, and proteins across epithelial cell sheets. Disruption of these junctions due to aberrant PDZ protein function is predicted, therefore, to cause leakiness of CSF, thus leading to hydrocephalus. The 13 PDZ domains of the MPDZ protein form a scaffold for several other tight junction components, such as claudins and angiomotin (AMOT) (8). AMOT is essential for maintaining junction integrity by targeting the PDZ-domain proteins Pals1/Patj and Par3 to tight junctions (9); however, no pathogenic variant in this gene has been reported to date in humans as related to hydrocephalus.

AMOT functions as an essential regulator of vessel formation during embryonic development. Aase et al. observed dilation and lagoon-like structures in brain vessels and yolk sacs of heterozygote AMOT-deficient mice embryos at day 10.5 (10). AMOT is a scaffolding tight junction protein that participates in multiple signaling pathways, such as the BMP signaling pathway (11). AMOT acts as an adaptor protein by interacting with the BMP type II receptor (BMPR2) and modulates apical BMP signaling in polarized cells. We have shown that siRNA-mediated AMOT knockdown causes less apical SMAD1/5 phosphorylation upon BMP stimulation and decreases resistance between cell junctions (11). Therefore, tight regulation of AMOT is required for the proper functioning of junctions.

Due to alternative exon splicing, 2 isoforms with different molecular weights are produced: the long AMOT130 and short AMOT80 isoforms (12). AMOT130 has an extended N-terminus necessary for BMPR2-AMOT interaction and contains L/P-PXY, which regulates the stability of the protein (13). L/P-PXY motifs are hubs for association of AMOT130 with several proteins (i.e., YAP/TAZ; ref. 14) and 3 members of the neural precursor cell–expressed, developmentally downregulated 4 (Nedd4) family of E3 HECT ubiquitin ligases, Nedd4, Nedd4-2 and Itch. Nedd4, Nedd4-2, and Itch recognize the L/P-PXY^106–109, 239–242, 284–287^ regions of AMOT130 via their WW domain and transfer ubiquitin molecules to the K481 residue (15). The RING finger protein 146 (RNF146) can ubiquitinate AMOT after it is poly-ADP ribosylated (PARsylated) by a poly (ADP-ribose) polymerase called tankyrase (TNKS) via recognition of the tankyrase-binding domain^77–84^ (TBD) (16). In addition to the specific domains, N-terminal amino acid residues are critical to determine the lifespan of the proteins (17). UBR4 is a member of the N-end rule pathway detecting the destabilizing N-terminal amino acid residues (N-degrons) of its target proteins, and it plays an important role in neurogenesis and the homeostasis of cell-surface proteins (18). Go et al. showed in a proximity-dependent biotin-identification study that the N-recognin UBR4 is near AMOT (19). However, to date, no known UBR4-mediated ubiquitination of AMOT has been shown experimentally.

**Figure 1:**
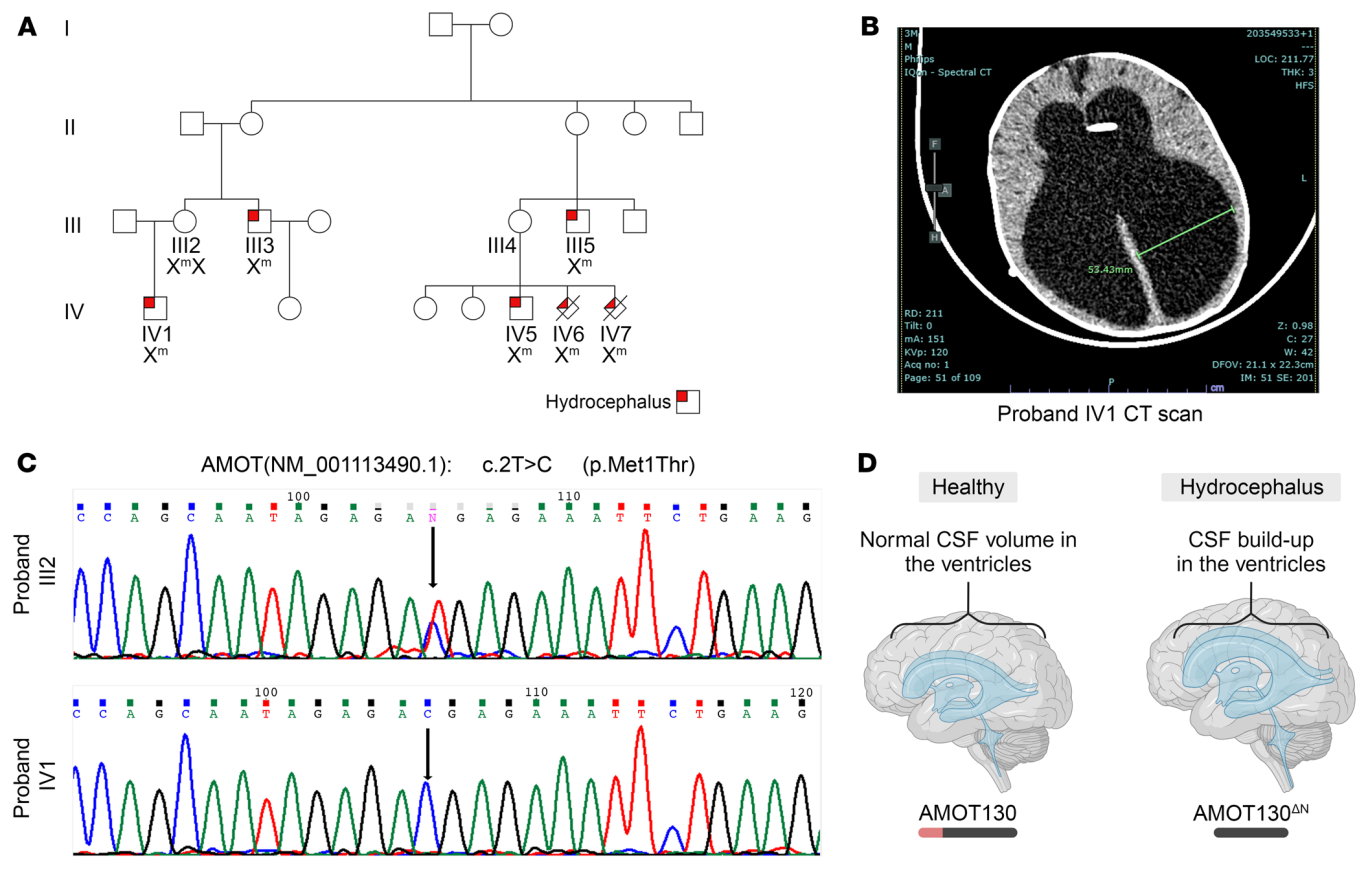
AMOT-linked congenital hydrocephalus presentation in the affected family. (**A**) The affected family pedigree. Upper-left red quadrant denotes affected by hydrocephalus. Genotype of the p.Met1Thr AMOT variant is given. (**B**) CT scan of the proband IV1 during shunt reinsertion at age 3 months. (**C**) Sequencing chromatogram of the patient and mother showing the variant in AMOT. (**D**) Representative scheme depicting cerebral ventricles of healthy individual designated AMOT130 (left) and of a patient with a hydrocephalus due to the p.Met1Thr variant in AMOT130^ΔN^ (right). CSF, cerebral spinal fluid.

Here, we describe 1 of the first diseases caused by an *AMOT* variant in humans (20), manifesting as isolated X-linked hydrocephalus (Figure 1D). We show that this variant causes an N-terminus deletion of AMOT, rendering the protein less accessible to the protein degradation machinery and thus increasing its stability. Abnormally high levels of the AMOT protein affect its proper function (i.e., disrupting the junctional properties in polarized epithelial cells), thereby providing the disease mechanism. Of clinical importance, affected individuals present with normal development into adulthood after early neonatal ventriculoperitoneal (VP) shunt insertion. This observation has radical consequences because it suggests that early treatment can maintain normal neurodevelopment, despite the ominous sonographic appearance of the brain during pregnancy.

## Results

### Family description and clinical phenotype of individuals affected by AMOT mutation.

Family A attended the genetic counseling clinic at Hadassah Ein Karem Medical Center after the diagnosis of their son (proband IV1) with congenital hydrocephalus (see family pedigree in Figure 1A). Family A displays a clear pattern of X-linked isolated hydrocephalus in the family, with 4 affected live-born male infants (III3, III5, IV5, and IV1) and 2 terminated male fetuses (IV6 and IV7 at 28 weeks and 32 weeks of gestation, respectively), due to hydrocephalus detected on MRI scans (Supplemental Figure 1, A and B; supplemental material available online with this article; https://doi.org/10.1172/JCI179438DS1). The inheritance is via unaffected women over 3 generations. The live-born hydrocephalic patients were treated with VP shunt insertion shortly after birth. All 4 are healthy and their neurodevelopment is normal, including the 2 adults (III3 and III5) who have graduated with higher education degrees. Carrier females are apparently normal, although subtle neuroradiological finding cannot be excluded, because none of the obligate carriers have undergone brain MRI.

The clinical presentation of hydrocephalus in this family is rather late, with routine ultrasound scan performed at gestational weeks 9 and 22 failing to detect any abnormality. During the pregnancy, proband IV1’s enlarged brain ventricles were first detected at week 29 by routine scan. Fetal brain MRI confirmed the finding and demonstrated normal brain structure and no parenchymal findings. The child was delivered at 38 weeks via cesarean section; at birth, his head circumference was 38 cm (>97th percentile). A postnatal brain MRI scan at 2 days of age disclosed enlarged brain ventricles and secondary thinning of the corpus callosum, with normal brain structure. Due to rapid head growth, the patient underwent a right-sided VP shunt insertion at 3 weeks, which slowed the head growth and initiated the attainment of typical developmental milestones. At an assessment with a clinical geneticist at the age of 2 months, the head circumference measured 42 cm (93rd percentile), and a depressed nasal bridge was noted, as was frontal bossing, but no other dysmorphic features or physical anomalies were detected. The clinical course was complicated by sudden growth of the child’s head circumference at 3 months (46 cm at admission), raising a suspicion of VP shunt malfunction. This was treated by VP shunt replacement (Figure 1B). The child was since followed up at the community clinic and normal attainment of developmental milestones has been reported.

### Clinical exome sequencing revealed a hemizygous single point mutation of AMOT.

Initial genetic investigation of proband IV1 showed normal chromosomal microarray analysis result. The family opted to continue genetic testing of proband IV1 via clinical exome sequencing (ES) at the Hadassah Medical Center deep-sequencing laboratory (EMQN certified). This analysis identified a single, likely pathogenic, X-linked variant, c.2T>C p.Met1Thr, in the *AMOT* gene (Hg19 ChrX:112066353 T>C) (Figure 1C). Using multiple pathogenicity–predicting software, the variant was predicted to be likely pathogenic, affecting the first coding methionine of the gene. The variant was tested by Sanger sequencing in the available 8 family members. The genotype of these individuals segregated with their disease state, supporting the association of the *AMOT* variant with the disease (Figure 1A). No pathogenic variants in AMOT have been identified in another family during screenings of patients.

### The variant of AMOT results in a truncated protein, AMOT130^ΔN^, with increased protein stability.

**Figure 2:**
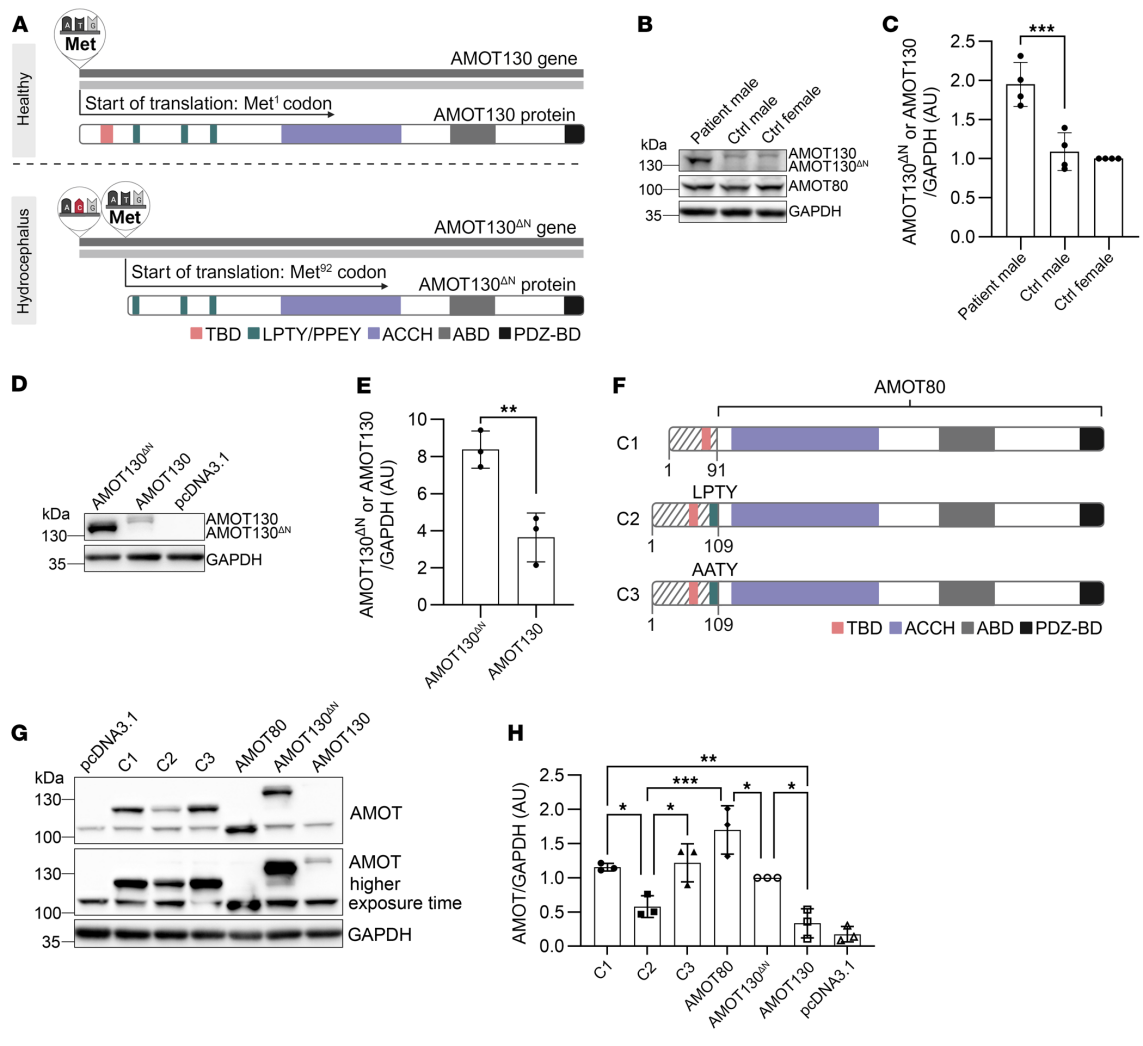
Single point mutation in the start codon of the *AMOT* gene leads to an in-frame shift to a new start codon (Met^92^) and thereby truncated AMOT130, herein called AMOT130^ΔN^, which presents with elevated protein levels. (**A**) Schematic representation of AMOT (upper panel) and AMOT130^ΔN^ (lower panel) genes and proteins. ABD, angiostatin binding domain; PDZ-BD, PDZ binding domain. (**B**) Representative Western blot indicating an approximately 10 kDa molecular weight shift of AMOT130^ΔN^ (first lane) compared with AMOT130 and increased protein level of AMOT130^ΔN^ in primary skin fibroblasts obtained from proband IV1 (male patient) compared with AMOT130 in a male individual acting as a control (ctrl) and a female individual acting as a control. No change in AMOT80 protein level was detected between the patient and control groups. (**C**) Quantification of AMOT130^ΔN^ and AMOT130 protein levels in patient/donor–derived primary skin fibroblasts by taking GAPDH as a housekeeping gene. *n* = 4 independent experiments; the SD is reported. ***P < 0.001, ordinary 1-way ANOVA with Tukey’s post hoc test. (**D**) Representative Western blot of overexpressed AMOT130^ΔN^, AMOT130, or pcDNA3.1 vector backbone control in epithelial cells (MCF7). (**E**) Quantification of overexpressed AMOT130^ΔN^ and AMOT130 protein levels in MCF7 cells. The housekeeping gene was GAPDH. The mean of 3 independent experiments with SD. **P < 0.01, unpaired *t* test. (**F**) Chimeric proteins scheme. (**G**) Representative Western blot of 3 independent experiments depicting overexpression of chimeric, AMOT130^ΔN^, and AMOT130 protein levels in MCF7 cells. (**H**) Quantification of chimeric, AMOT130^ΔN^, and AMOT130 protein levels of 3 independent experiments (SD reported). *P ≤ 0.05, **P ≤ 0.01, ***P ≤ 0.001, ordinary 1-way ANOVA with Tukey’s post hoc test.

The missense variant in the start codon (ATG) of *AMOT* was speculated to cause an in-frame shift of the start of translation to the next methionine, generating a protein, AMOT130^ΔN^, that is shorter at the N-terminus by 91 amino acids (Figure 2A). This protein was expected to be about 10 kDa smaller. To confirm the translation of AMOT130^ΔN^ in patient/donor–derived cells (the patient being proband IV1), we subjected healthy (male and female) and patient (male) skin fibroblast cell lysates to Western blotting by using an AMOT antibody that recognizes the C-terminus of AMOT. Thereby, we proved the translation and truncation of AMOT130^ΔN^ by observing a 10 kDa smaller protein (Figure 2B). Moreover, we observed a strong increase of AMOT130^ΔN^ protein level compared with AMOT130 in male and female individuals acting as controls (Figure 2, B and C), even though gene expression levels were not increased (Supplemental Figure 2A). The protein level of the short isoform AMOT80 was not affected in the patient (Figure 2B and Supplemental Figure 2B).

Based on the patient’s phenotype, which was confined to the brain, we expected that brain-specialized epithelial cells, the ependymal cells (21), would express the impact of the *AMOT* variant. We thus confirmed the increase of AMOT130^ΔN^ protein levels in an epithelial cell model. To this end, we overexpressed AMOT130 or AMOT130^ΔN^ in Michigan Cancer Foundation-7 (MCF7) cells and found that, consistent with the data from fibroblasts, AMOT130^ΔN^ resulted in a 10 kDa smaller protein and significantly higher protein levels as compared with AMOT130 (Figure 2, D and E, respectively). The observation that AMOT130^ΔN^ protein levels are increased by 1.8- or 3.5-fold, respectively, in both fibroblasts and epithelial cells led us to hypothesize that the first 91 amino acids of AMOT are critical for regulating AMOT protein stability. Therefore, we generated 3 chimeric proteins (Figure 2F): (a) chimeric protein 1 (C1), a fusion protein of the first 91 amino acids of AMOT130 and AMOT80; (b) because the LPTY motif is essential for the recognition of AMOT by ubiquitin ligases (15), we included the LPTY motif to C1 to generate chimeric protein 2 (C2); and (c) we mutated the LPTY in C2 to AATY to obtain chimeric protein 3 (C3) to confirm the effect of LPTY motif presence in case of any change in protein level. Strikingly, adding 91 amino acids to the N-terminus of AMOT80 decreased the protein level of C1 compared with AMOT80 (Figure 2, G and H). The C2 protein level was less than that of C1, C3, and AMOT80, highlighting the importance of the first 91 amino acids and the LPTY motif in protein stability and degradation (Figure 2, G and H).

Taken together, we have shown that the p.Met1Thr variant in the start codon of the *AMOT* gene in patients with hydrocephalus leads to an in-frame shift to the next ATG, resulting in a protein (AMOT130^ΔN^) of smaller size but increased protein stability, due to the loss of the N-terminal 91 amino acids.

### The stability of AMOT130^ΔN^ is changed due to the deletion of the TBD and altered N-degron degradation signaling.

**Figure 3:**
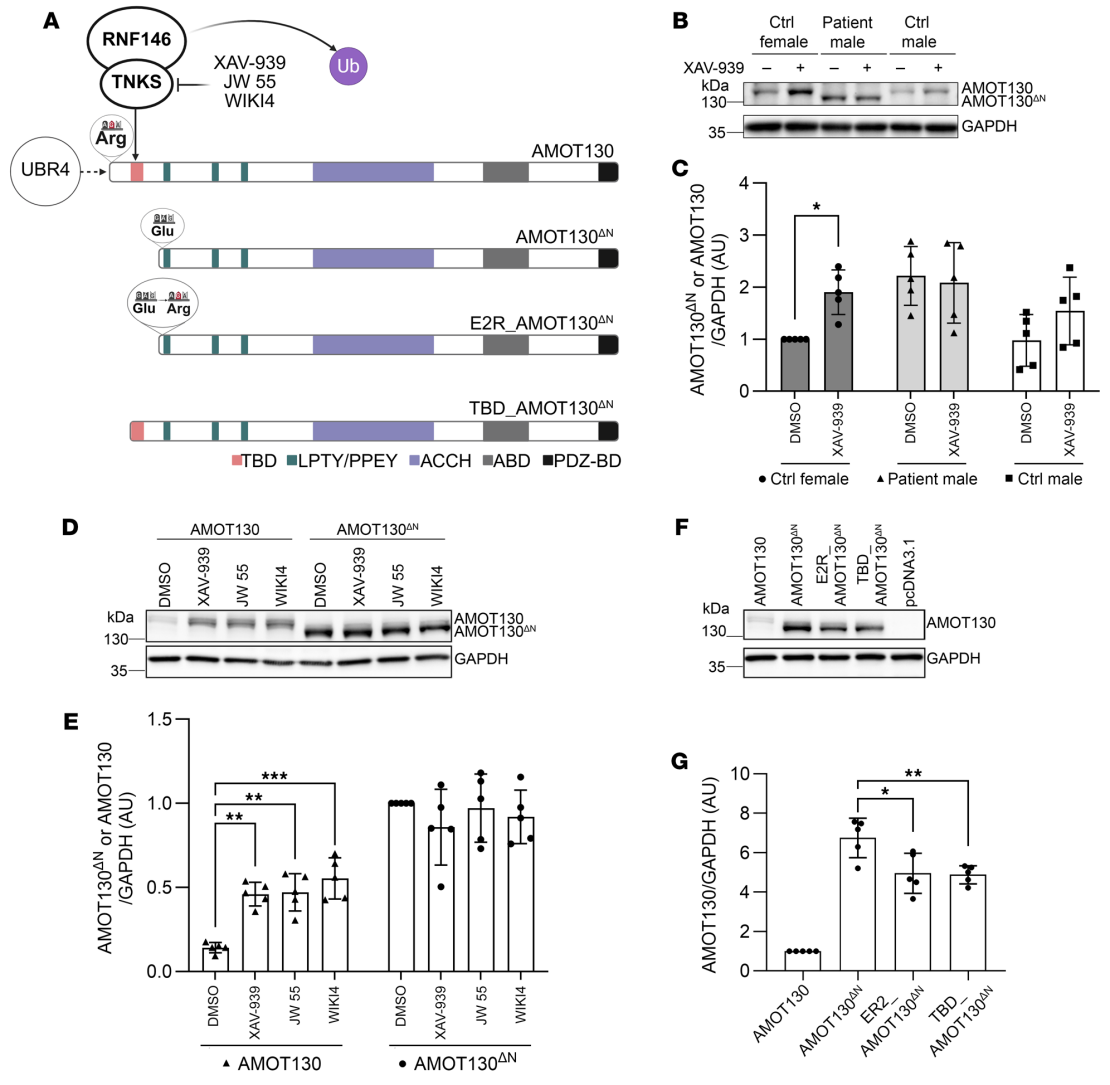
Elevated AMOT130^ΔN^ protein level is caused by TBD loss and might be linked to altered N-degron property. (**A**) Illustration of the second position amino acid of AMOT130, AMOT130^ΔN^, and rescue construct E2R_AMOT130^ΔN^; TNKS inhibition via XAV-939; and RNF146-mediated ubiquitylation of AMOT130. (**B**) Representative Western blot depicting AMOT130^ΔN^ and AMOT130 protein levels after TNKS inhibition by XAV-939 in primary skin fibroblasts derived from a female control, a male patient, or a male control. (**C**) Quantification of 5 independent Western blots shows that the protein levels of AMOT130 in male and female control primary skin fibroblasts are increased but AMOT130^ΔN^ level is not affected in patient primary skin fibroblasts upon XAV-939 treatment. The SD is reported. *P < 0.05, 2-way-ANOVA with Šídák’s multiple comparisons tests. (**D**) Representative Western blot depicting overexpressed AMOT130^ΔN^ and AMOT130 protein levels after XAV-939, JW 55, and WIKI4 (10 μM, for 12 hours) addition to MCF7 cells. (**E**) Quantification of 5 independent Western blots shows that overexpressed AMOT130 protein level is increased, but overexpressed AMOT130^ΔN^ protein level is unaffected after XAV-939, JW 55, and WIKI4 treatment in MCF7 cells. The SD is reported. **P ≤ 0.01, ***P < 0.001, 2-way ANOVA with Šídák’s multiple comparisons tests. (**F**) Representative Western blot demonstrating protein levels of AMOT130, AMOT130^ΔN^, E2R_AMOT130^ΔN^, and TBD_AMOT130^ΔN^. E2R_AMOT130^ΔN^ mimics the destabilized N-terminus of AMOT130 by 1 amino acid substitution in the second position: glutamic acid to arginine (E2R) in AMOT130^ΔN^. TBD_AMOT130^ΔN^ contains TBD^77–84^ of wild-type AMOT130 at the N-terminus of AMOT130^ΔN^. (**G**) Quantification of 5 independent Western blots shows that the protein level of overexpressed E2R_AMOT130^ΔN^ and TBD_AMOT130^ΔN^ is significantly decreased compared with AMOT130^ΔN^ but is still higher than AMOT130. The SD is reported. *P ≤ 0.05, **P ≤ 0.01, 1-way ANOVA with Dunnett’s multiple comparisons test. (AMOT130^ΔN^ was taken as a control group.)

The higher protein level of AMOT130^ΔN^ compared with AMOT130 directed us to investigate potential changes in the degradation machinery of AMOT. One of the predicted TNKS binding domains, TBD^77–84^, is lost in AMOT130^ΔN^ (Figure 3A). The wild-type AMOT TBD^77–84^ domain is recognized and PARsylated by TNKS, leading to its degradation through RNF146 (16). Thus, the loss of the TBD motif might disrupt the interaction between AMOT130^ΔN^ and the TNKS-RNF146 axis, ultimately leading to the stabilization of the mutant AMOT. To test our hypothesis, we treated fibroblasts with XAV-939, a small-molecule inhibitor against TNKS. As expected, after TNKS inhibition, AMOT130 levels increased in control fibroblasts (significantly in fibroblasts from female donors), whereas AMOT130^ΔN^ protein levels did not change in patient-derived fibroblasts in which the TBD^77–84^ is lost (Figure 3, B and C). To further validate the effect of TNKS inhibition, we treated MCF7 cells expressing either AMOT130 or AMOT130^ΔN^ with 3 different small TNKS inhibitors: XAV-939, JW 55, or WIKI4. In line with the results in fibroblasts, treatment with TNKS inhibitors significantly increased the level of AMOT130 in cells expressing it, whereas the levels of AMOT130^ΔN^ remained unchanged (Figure 3, D and E).

To further demonstrate that loss of TBD^77–84^ might contribute to the elevated levels of AMOT130^ΔN^, we generated a construct, called TBD_AMOT130^ΔN^, incorporating TBD at the N-terminus of AMOT130^ΔN^ (Figure 3A). TBD_AMOT130^ΔN^ or AMOT130^ΔN^ constructs were overexpressed in MCF7 cells. Quantifying protein levels revealed that those of TBD_AMOT130^ΔN^ were approximately 30% lower than AMOT130^ΔN^ levels (Figure 3F, lanes 2 and 4, and Figure 3G). Thus, we concluded that the loss of TBD^77–84^ is partially responsible for the elevated level of mutant AMOT.

The loss of the TBD is not the only change in the N-terminus of AMOT130^ΔN^ that might play a role in its altered stability. The second amino acid residue of AMOT130 is arginine (R); in AMOT130^ΔN^ this residue is glutamic acid (E). Arginine at the second position with intact methionine has been shown to be more susceptible to degradation by UBR family E3 ligase (22). Therefore, we hypothesized that UBR4, an N-end rule pathway member and possible ubiquitin ligase of AMOT (19), might have an altered interaction with the mutant isoform. To test this idea, we generated a construct of AMOT130^ΔN^, which contains E to R substitution (E2R_AMOT130^ΔN^; Figure 3A), to potentially restore the affinity of UBR4 to the mutant isoform and, therefore, decrease its protein level. Overexpressed E2R_ AMOT130^ΔN^ protein levels were 1.4-fold significantly lower (*P* = 0.012) than AMOT130^ΔN^ levels in MCF7 cells (Figure 3, F and G). This indicates a potential rescue in UBR4 recognition of AMOT130^ΔN^ by reintroducing a destabilized amino acid residue in E2R_ AMOT130^ΔN^. Given that the decreased protein levels of E2R_ AMOT130^ΔN^ could not reach the level of wild-type AMOT130, we think the loss of the N-degron property is a secondary factor to the absence of TBD^77–84^ that accounts for the elevated protein level of AMOT130^ΔN^.

Next, we performed siRNA-mediated gene silencing of UBR4 in patient and control skin fibroblasts. We expected to observe an increase only in the AMOT130, not in AMOT130^ΔN^, protein level under UBR4 knockdown conditions. However, siRNA-mediated UBR4 knockdown revealed that both AMOT130^ΔN^ and AMOT130 protein levels tended to decrease in patient and control fibroblasts (Supplemental Figure 3, A–C), indicating again that altered interaction of UBR4 does not solely account for the high AMOT130^ΔN^ level. Hunt et al. (23) described UBR4 protein targets via tandem mass tag mass spectroscopy and RNA sequencing in mice tibialis anterior muscle by comparing UBR4-knockdown and control samples. According to tandem mass tag mass spectroscopy, UBR4 knockdown upregulated NEDD4 and NEDD4-2 protein levels 0.8- and 0.4-fold, respectively (Supplemental Figure 3D), whereas mRNA levels of the same proteins were not significantly regulated (Supplemental Figure 3E). This elevated level of the AMOT upstream regulators upon UBR4 knockdown might explain the decrease of AMOT protein level via enhanced degradation processes.

In summary, we showed that the loss of 91 amino acids at the N-terminus of AMOT130 alters the stability of AMOT protein by affecting the degradation machinery due to the loss of the TBD and changes in its N-degron characteristics.

### AMOT130^ΔN^ interacts with BMPR2 and localizes at the plasma membrane.

**Figure 4:**
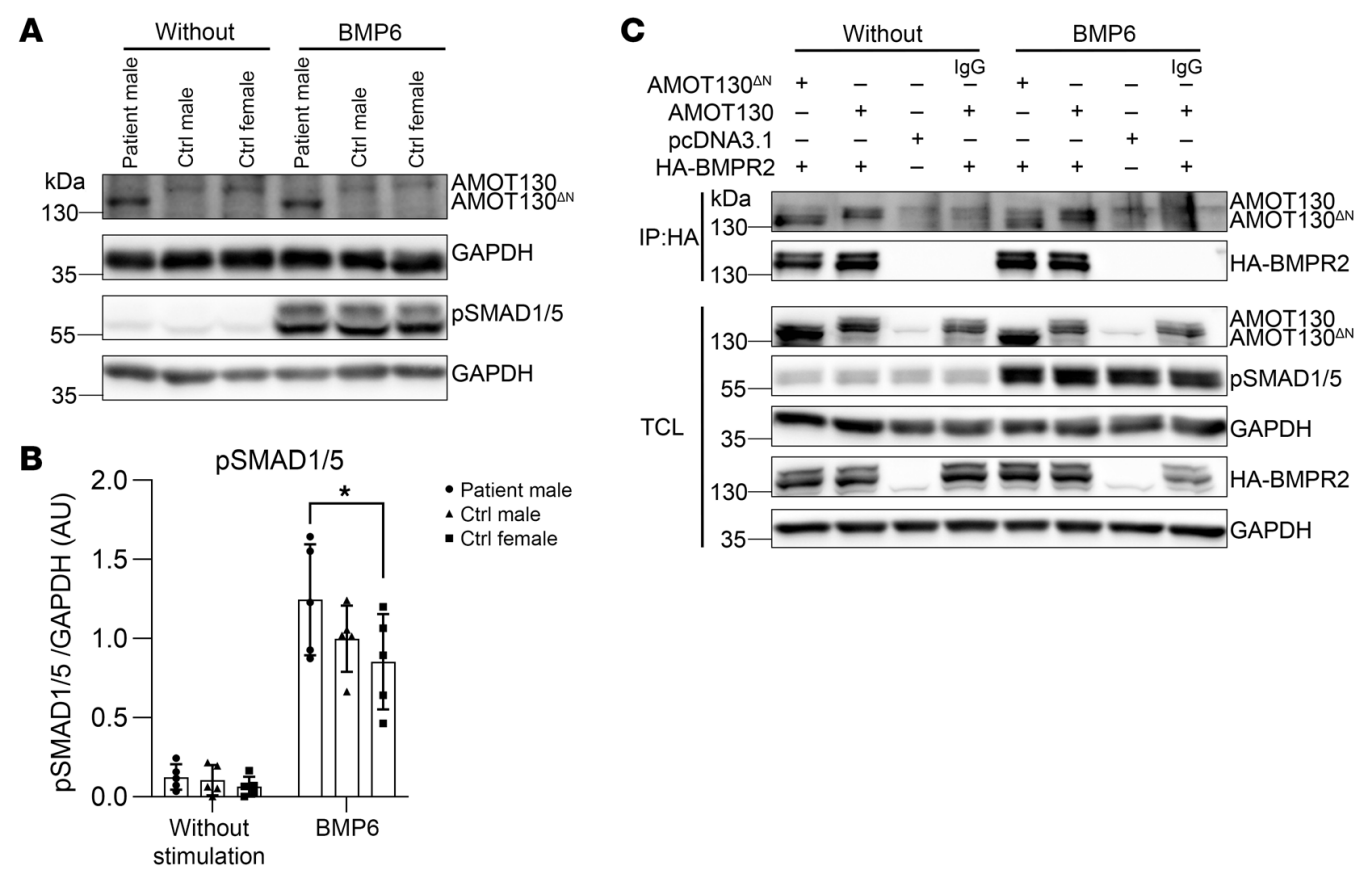
SMAD1/5 phosphorylation level after BMP6 stimulation is slightly higher in AMOT130^ΔN^-expressing patient cells than control fibroblasts, and AMOT130^ΔN^ still interacts with BMPR2. (**A**) Representative Western blot demonstrating AMOT130^ΔN^ and pSMAD1/5 proteins upon 30-minute BMP6 (10 nM) stimulation in primary skin fibroblasts derived from male patient, male control, or female control. (**B**) Quantification of pSMAD1/5 levels upon BMP6 stimulation (stim) in male patient, male control, and female control skin fibroblasts. SDs from 5 independent experiments are reported. *P < 0.05, 2-way ANOVA with Tukey’s multiple comparisons test. w/o, without. (**C**) Representative Western blot showing AMOT130^ΔN^-BMPR2 (lanes 1 and 5) and AMOT130-BMPR2 (lanes 2 and 6) interaction via immunoprecipitating HA-BMPR2 and detecting AMOT130^ΔN^ and AMOT130 in HEK293T cells.

AMOT participates in many signaling pathways at different stages of transducing the signal from the cell surface to the nucleus. The BMP signaling pathway is 1 of the most crucial pathways in which AMOT acts as an adaptor protein. Brunner et al. demonstrated that AMOT knockdown decreases SMAD1/5 phosphorylation in polarized cells (11). To investigate the effect of AMOT130^ΔN^ mutation on BMP pathway signaling, we analyzed the SMAD1/5 phosphorylation level upon BMP6 stimulation in patient and healthy fibroblasts, and in MCF7 cells expressing AMOT130^ΔN^ or overexpressing AMOT130. BMP6 stimulation demonstrated that SMAD1/5 phosphorylation was slightly higher in patient-derived fibroblasts than control fibroblasts (Figure 4, A and B), whereas no significant change was observed between AMOT130^ΔN^- or AMOT130-expressing MCF7 cells (Supplemental Figure 4, A and B). As previously shown, AMOT exerts its adaptor and BMP signaling functions primarily from the apical compartment of the polarized cells (11). To test whether this unique apical modulating BMP signaling effect of AMOT is affected in AMOT130^ΔN^, we stimulated polarized AMOT130^ΔN^- or AMOT130- expressing MCF7 cells cultured in Transwell plates from the apical or basal side with BMP6. SMAD1/5 phosphorylation was slightly higher in apically stimulated AMOT130^ΔN^- versus AMOT130-expressing MCF7 cells (Supplemental Figure 4, C and D), whereas BMP6 stimulation from the basal side resulted in a subtle decrease in SMAD1/5 phosphorylation in AMOT130^ΔN^-transfected MCF7 cells (Supplemental Figure 4E).

The extended N-terminus of AMOT130 is a hub that interacts with various proteins and regulates cellular dynamics (e.g., cytoskeleton bundling and YAP/TAZ signaling; ref. 24). We have also shown that only AMOT130, but not AMOT80, interacts with BMPR2 (11). Because 91 amino acids are lost at the beginning of the N-terminus in AMOT130^ΔN^, we investigated whether AMOT130^ΔN^ still interacts with BMPR2. HA-tagged BMPR2 (HA-BMPR2) was coexpressed with either AMOT130^ΔN^ or AMOT130 in human embryonic kidney (HEK293T) cells to be followed by coimmunoprecipitation (CO-IP) with or without BMP6 stimulation. Pulldown of BMPR2 via HA-antibody results in coprecipitated AMOT130, which confirms the AMOT130-BMPR2 interaction. Strikingly, we observed that AMOT130^ΔN^ can interact with BMPR2 (Figure 4C). By showing the interaction between AMOT130^ΔN^ and BMPR2, we narrowed down the interaction site to the region between amino acid 92 and amino acid 409.

**Figure 5:**
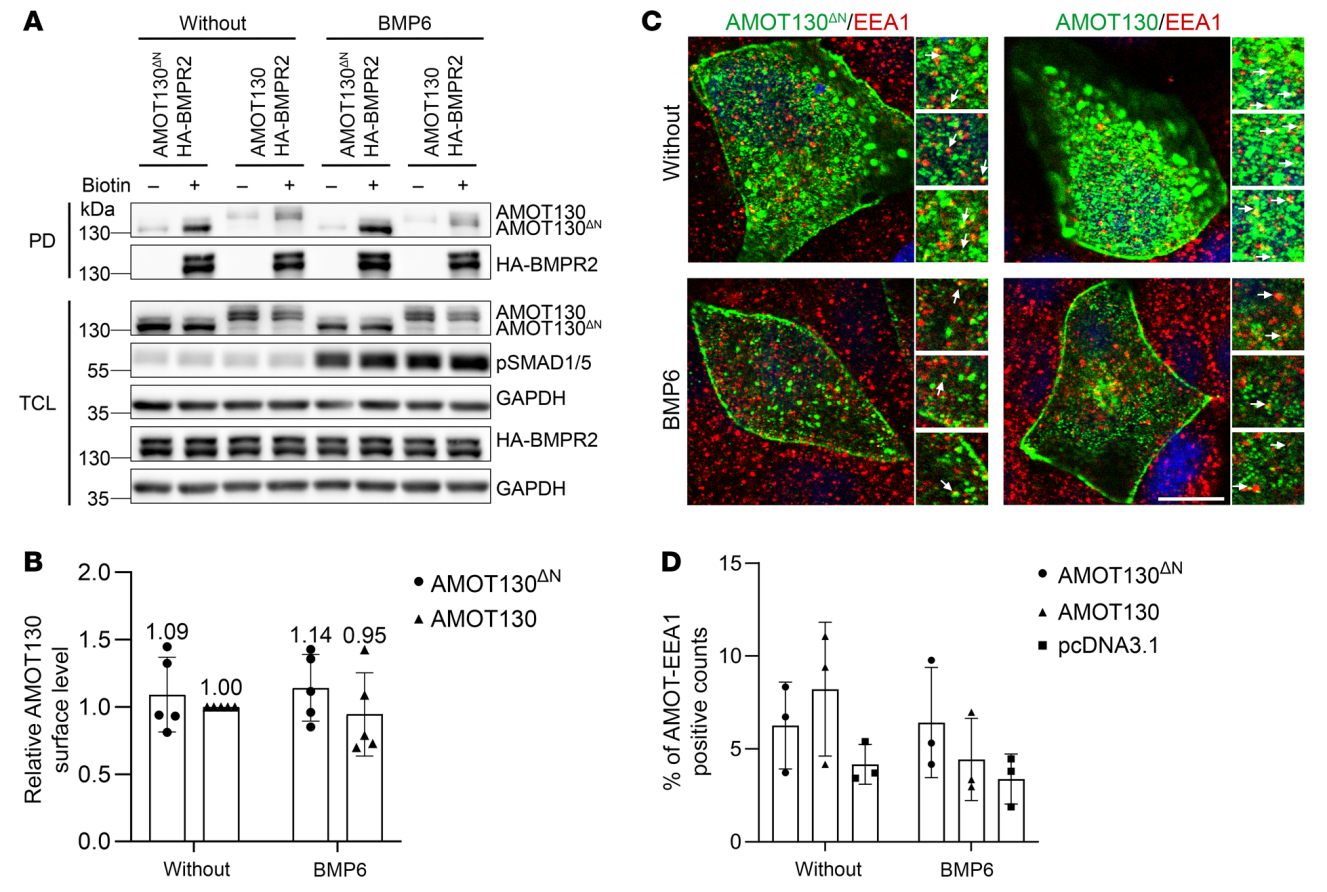
AMOT130^ΔN^ localizes at the cell surface as AMOT130. (**A**) Western blot analysis depicting membrane levels of AMOT130^ΔN^, AMOT130, and BMPR2 after surface biotinylation assay in HEK293T cells. PD, pull down, TCL, total cell lysate. (**B**) Quantification of pSMAD1/5 levels upon BMP6 stimulation in male patient, male control, or female control skin fibroblasts. (**C**) IF imaging results illustrating AMOT130^ΔN^/AMOT130 (green) and EEA1 (red) colocalization in yellow colors shown by arrows in insets. Scale bar: 10 μm. (**D**) Quantification of AMOT-EEA1 colocalization.

We have shown that activation of the BMP cascade triggers AMOT internalization, probably into endosomal structures (11). To investigate the localization of AMOT130^ΔN^ at the cell membrane and endosomal level after BMP6 stimulation, we performed a surface biotinylation assay and early endosome antigen 1 (EEA1)-AMOT costaining. HEK293T cells were transfected with HA-BMPR2 and AMOT130^ΔN^ or AMOT130 constructs. Then, biotinylated surface proteins, including AMOT and BMPR2, were pulled down by streptavidin beads after BMP6 stimulation. After 30 minutes of BMP6 stimulation, AMOT130^ΔN^ and AMOT130 localization could still be observed at the cell membrane (Figure 5A), and their surface levels were comparable upon BMP6 stimulation (Figure 5, A and B). EEA1-AMOT colocalization analyses revealed that early endosomes were positive for both isoforms of the AMOT (Figure 5C and Supplemental Figure 5A, depicted with arrows in the inlets). According to the quantification of AMOT-EEA1 colocalization, BMP6 stimulation triggered more endocytosis of AMOT130^ΔN^ compared with AMOT130 (Figure 5D), probably as a compensation mechanism.

Taken together, we demonstrated that increased AMOT130^ΔN^ protein level has only minor effects on the SMAD signaling pathway. However, we revealed that BMPR2 and AMOT130^ΔN^ still interact, narrowing the interaction site in AMOT to between amino acid 92 and amino acid 409. We showed that AMOT130^ΔN^ localized at the plasma membrane and that early endosomal localization of AMOT130^ΔN^ is increased compared with AMOT130, which might be a compensation mechanism to balance AMOT130^ΔN^ levels.

### AMOT130^ΔN^ subcellular localization is predominantly apical as AMOT130 in polarized cells; however, AMOT130^ΔN^-expressing epithelial cells’ barrier integrity is lower than that of AMOT130-expressing cells.

**Figure 6:**
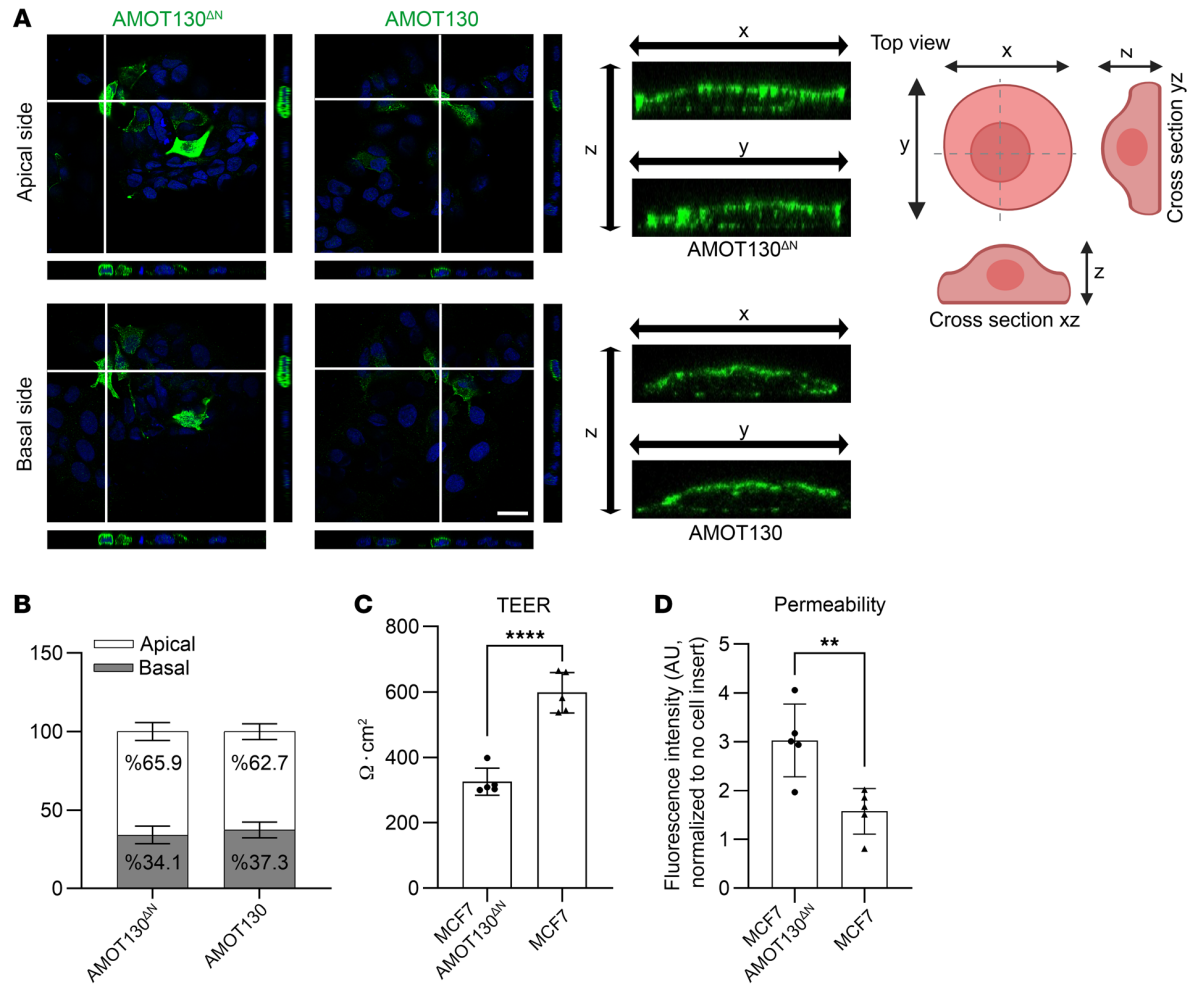
Localization of AMOT130^ΔN^ is still predominantly at the apical membrane, as shown for AMOT130 in epithelial cells, but AMOT130^ΔN^ expression leads to barrier integrity loss. (**A**) Representative top view and Z-stack IF images of MCF7 cells with AMOT130^ΔN^/AMOT130 (green) staining. Nuclei were stained with DAPI (blue). Representative *xz* and *yz* cross sections of AMOT130^ΔN^- or AMOT130-expressing MCF7 cells, showing the localization of AMOTs at the apical and basal sides (the 4 images in the middle panel). Dashed lines show the schematically represented cross-sections (drawing in the right panel). Scale bar: 25 μm. (**B**) Quantification of AMOT130^ΔN^ and AMOT130 apical versus basal localization from 3 independent IF staining experiments. (**C**) TEER measurement of 5 independent experiments shows significantly decreased barrier integrity in AMOT130^ΔN^-expressing MCF7 cells compared with wild-type MCF7 cells, cultured for 6 days in Transwell plates. The SD is reported. ****P < 0.0001, unpaired *t* test. (**D**) Fluorescence intensity measurement of 5 independent experiments shows significantly increased permeability of the cell monolayer established by AMOT130^ΔN^-expressing MCF7 cells compared with AMOT130-expressing cells cultured for 6 days in Transwell plates. Permeability assays were performed by adding 4 kDa FITC-dextran from the apical side, and fluorescence intensity was measured in the collected basal medium after 4 hours. **P < 0.01, unpaired *t* test.

AMOT normally localizes at the apical membrane and cell-cell junctions of polarized cells (9, 11). To investigate AMOT130^ΔN^ localization in patient fibroblasts, we stained AMOT via immunofluorescence (IF) staining. To label the apical part of the cells, we carried out protein kinase C-ζ (PKC-ζ) and AMOT costaining. No dramatic change of localization was observed between the patient and control fibroblasts. Endogenous AMOT130^ΔN^ and AMOT130 both localized in the cytoplasm and the nucleus, because these cells do not show apical-basal polarization (Supplemental Figure 5B). Therefore, we investigated a potential mislocalization of AMOT130^ΔN^ in our epithelial cell model after expressing corresponding constructs. AMOT130^ΔN^ and AMOT130 localized to about 66% and 63% to the apical side, respectively (Figure 6, A and B, and Supplemental Figure 5C). Even though both variants are expressed at similar percentages at the apical plasma membrane of MCF7 cells, transepithelial resistance (TEER) measurements demonstrated a 50% decrease in the barrier integrity in AMOT130^ΔN^-expressing cells compared with wild-type MCF7 cells (Figure 6C and Supplemental Figure 5D). In line with the TEER measurement, a permeability assay with 4 kDa FITC-labeled dextran indicated a significant increase of fluorescence intensity (1.9-fold) in AMOT130^ΔN^-expressing cells (Figure 6D and Supplemental Figure 5D).

In brief, we have shown that AMOT130^ΔN^ mutation did not cause changes in the subcellular distribution of the protein in epithelial cells. Still, AMOT130^ΔN^ expression caused a dramatic decrease in barrier integrity, as indicated by decreased TEER values and increased permeability of the cell layer compared with wild-type AMOT130-expressing cells, pointing out the functional consequences of this novel mutation.

The apparent change observed in the mutant AMOT is its elevated protein level compared with the wild-type AMOT130. To mimic this effect of mutant AMOT in TEER and permeability assays, we applied TNKS inhibitor to the cells (for 48 hours), which resulted in elevated levels of wild-type AMOT130 (Supplemental Figure 5E). Cells treated with XAV-939 had a 20% decrease in TEER values (Supplemental Figure 5F). Consistent with the TEER results, the permeability of cells treated with XAV-939 increased by 1.5-fold compared with DMSO-treated cells (Supplemental Figure 5G). Overall, our findings demonstrate that elevated levels of AMOT130 and AMOT130^ΔN^ disrupt the cell-cell junction integrity.

### Overexpression of AMOT130 and AMOT130^ΔN^ in human brain endothelial cells destabilizes cell-cell integrity.

**Figure 7:**
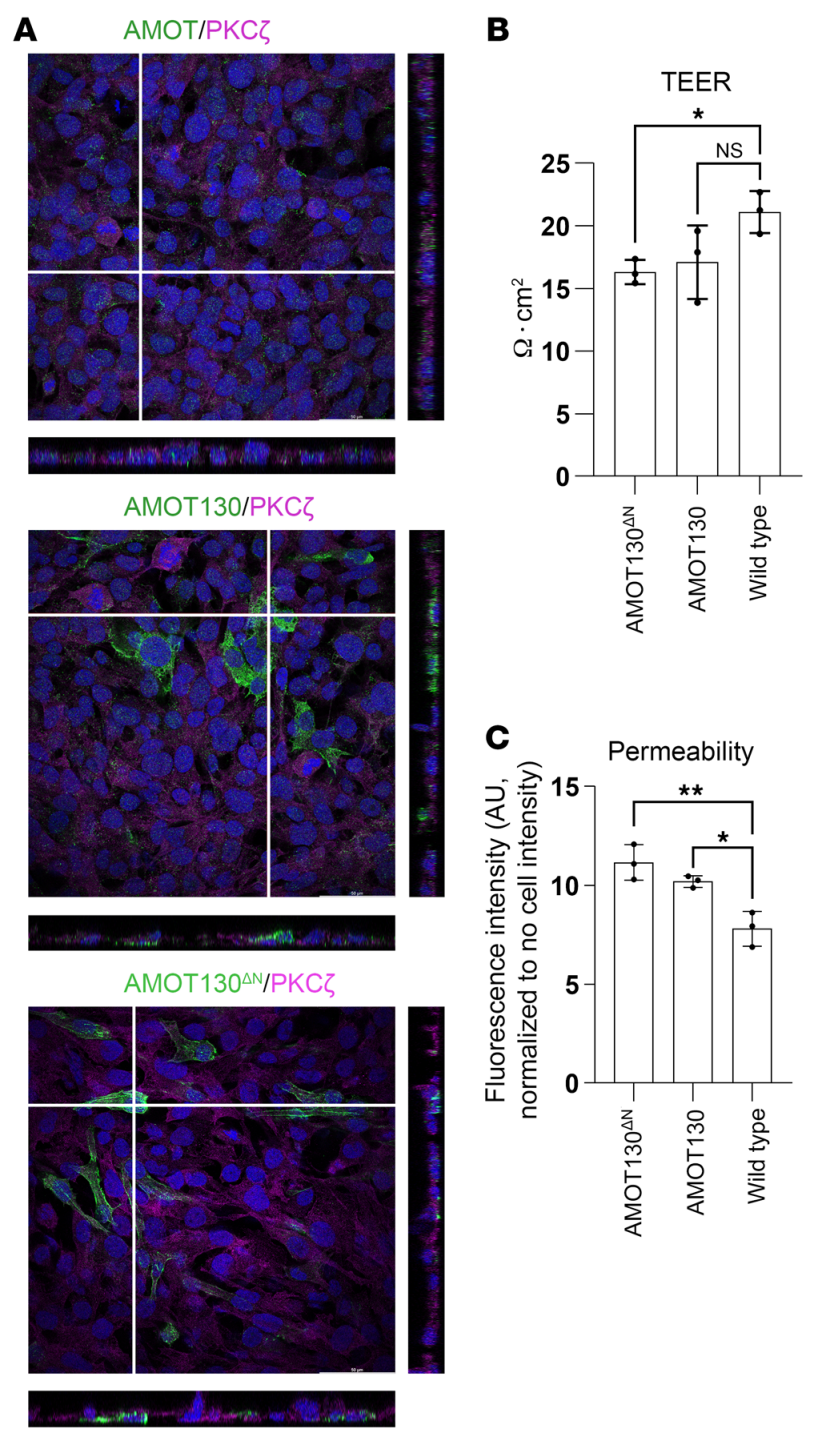
AMOT130^ΔN^ expression and AMOT130 overexpression disrupt the cell-cell barrier integrity in hCMECs. (**A**) AMOT and PKCζ costaining in hCMECs without overexpression (wild-type; upper image), or after AMOT130 (middle image) or AMOT130^ΔN^ (bottom image) overexpression. Nuclei were stained with DAPI and are shown in blue. (**B** and **C**) TEER measurement (**B**) and permeability assays (**C**) with AMOT130^ΔN^, AMOT130 overexpressing, or wild-type hCMECs. Cells were cultured for 4 days in Transwell plates inserted in growth medium. After completing the TEER measurement, 70 kDa FITC-dextran was added to the apical chamber of the Transwell plates for 4 hours for permeability assay. The fluorescence intensity of the collected basal medium was measured. The SD is reported. *P ≤ 0.05, **P ≤ 0.01, ordinary 1-way ANOVA, Dunnett’s multiple comparison test.

AMOT has critical functions in endothelial migration and angiogenesis (10, 25). Even though the brain vasculature of patients bearing AMOT130^ΔN^ was not dramatically different in computer tomography and ultrasound scans, magnetic resonance angiography was not carried out, which could reveal detailed information. However, dilated brain vessels were observed in AMOT-deficient mice embryos (10), indicating the importance of AMOT expression levels. Therefore, we overexpressed AMOT130^ΔN^ or AMOT130 in human cerebral microvascular endothelial cells (hCMECs) to compare changes in the localization of AMOT and to study cell-cell junction integrity. Surprisingly, AMOT did not localize specifically to the apical side in wild-type hCMECs (Figure 7A, upper image). AMOT130 and AMOT130^ΔN^ distributed to both the apical and basal sides of transfected cells (Figure 7A, middle and below image, respectively). Cell-cell junction stability was disrupted significantly in AMOT130^ΔN^-expressing and in AMOT130-overexpressing hCMECs. Compared with the wild-type hCMECs, TEER measurement revealed a 23% decrease in junctional integrity in AMOT130^ΔN^-expressing cells and a 20% reduction in AMOT130-overexpressing hCMECs (Figure 7B and Supplemental Figure 5E). Correspondingly, cell junction permeability, assessed by 70 kDa FITC-dextran accumulation in the basal part of the Transwell plates, increased by 40% in AMOT130^ΔN^-expressing cells and 30% in AMOT130-overexpressing hCMECs (Figure 7C and Supplemental Figure 5H). These data suggest elevated protein levels of either mutant or wild-type AMOT130 disrupts cell-cell junction dynamics, further supporting the hypothesis that aberrant AMOT protein expression contributes to the pathogenesis of hydrocephalus in affected patients.

## Discussion

We describe here 4 male patients with congenital hydrocephalus who all are from a single family. By ES, we found in these patients a human disease-causing variant in *AMOT*. This gene resides on the X chromosome, thus underlying X-linked inheritance of the disease in the family. *AMOT* encodes a tight junction protein, and its malfunction is expected to perturb barrier permeability of polarized cells, in agreement with the finding of congenital hydrocephalus in the patients. Until now, variants in only 1 chromosome of the X gene, *L1CAM*, were associated with congenital hydrocephalus (24). Unlike patients hemizygous for *L1CAM* pathogenic variants, who have additional radiological findings such as aqueductal stenosis and agenesis of the corpus callosum with resultant psychomotor morbidity, the 4 patients hemizygous for the *AMOT* variant developed normally after early VP shunt insertion. We have shown that the identified *AMOT* variant, p.Met1Thr (AMOT130^ΔN^), is associated with the production of a shortened AMOT protein, lacking its first 91 amino acids at the N-terminus.

AMOT is a scaffolding protein that localizes exclusively at the apical membrane of polarized cells. Because ependymal cells are specialized epithelial cells of the cerebral ventricles, proper AMOT localization and expression should be tightly regulated in these cells. Wells et al. (9) demonstrated that AMOT-overexpressing, polarized, Madin-Darby canine kidney cells (epithelial cells) relocalize Par-3 and Pals1 junction proteins from tight junctions to endocytic vesicles, thereby inducing loss of barrier integrity. Moreover, Wells et al. stated that AMOT levels must exceed a certain threshold to trigger the internalization of junction proteins. Strikingly, we observed that the expression level of the mutant AMOT protein, AMOT130^ΔN^, is dramatically elevated. Mechanistically, we showed that the loss of the TBD domain and N-degron property, both residing within the missing N-terminus, increases the protein’s stability by perturbing the AMOT130^ΔN^ degradation pathway.

Protein degradation plays a central role in the maintenance of physiological protein levels in cells. To achieve proteostatic balance, this process has to be firmly regulated in a spatiotemporal manner (26). Ubiquitylation is a control mechanism to ensure proper localization of the proteins. Ubiquitinylated plasma membrane proteins are marked for either degradation or recycling back to the plasma membrane or the Golgi apparatus (27). Any alteration or disruption of protein degradation can lead to various pathological conditions such as neurodegenerative and autoimmune disorders or cancer development (28, 29). In line with this knowledge, we have shown here that the increased stability of the newly identified AMOT mutant is 1 of the crucial reasons for development of congenital hydrocephalus.

Subcellular localization of Amot modulates Hippo signaling pathway activation during preimplantation (30, 31) and migration of visceral endoderm (32) in mouse embryos. Amot is preferentially distributed to the apical domain of the trophectoderm layer in blastocysts, where its localization is regulated by Rho-associated protein kinase. This distribution leads to the suppression of the Hippo pathway. In contrast, in the inner cell mass, Amot is concentrated at cell junctions, where it facilitates the activation of the Hippo pathway. Phosphorylation of AMOT at the S175 residue prevents its interaction with actin, promoting the activation of the Hippo pathway through enhanced AMOT-Lats binding stability (33). Thereby, the extended N-terminus of AMOT plays a crucial role in determining AMOT’s subcellular localization. Even though 91 amino acids have been lost in the AMOT130^ΔN^ mutant, the phosphorylation site, S175, and actin and YAP binding domains (33) are still present. Moreover, the AMOT coiled-coil homology (ACCH) domain of AMOT, which also determines its membrane localization, is unaffected by the herein described mutation (34). Therefore, the implantation of the mutant-carrying embryos into the uterus is not impaired. In addition, we revealed that AMOT130^ΔN^ localization is predominantly at the apical side, indicating that AMOT130^ΔN^ can still participate in and modulate different signaling cascades (e.g., the Hippo and BMP signaling pathways).

The BMP pathway involves neural induction and patterning of the central nervous system during embryonic development (35). BMP ligand expression has a complex spatiotemporal pattern during development. Different BMP ligands (e.g., BMP-2,-4,-5,-6, and -7) are expressed in the cerebral cortex, hippocampus, brain stem, and cerebellum (36). Differentiation of ependymal cells from radial glial cells, a subset of embryonic stem cells, is suppressed by BMP signaling in embryonic mouse telencephalic development (37). Additionally, the ciliogenesis of ependymal cells, which is essential for CSF circulation in the ventricles, is inhibited by BMP signaling (38).

Regulation of BMP signaling is critical for proper embryonic development of the central nervous system. By considering AMOT adaptor function in BMP signaling, we investigated in this study if mutated AMOT alters the BMP signaling dynamics. We showed that a higher level of AMOT130^ΔN^ causes a subtle increase in pSMAD1/5 levels in patient cells upon BMP6 stimulation. In addition, the biotinylation assay revealed that AMO130^ΔN^ localizes at the plasma membrane, as AMOT130 does. These findings suggest that AMOT130^ΔN^ and BMPR2 still interact, and AMOT130^ΔN^ has no dramatic effect on the BMP pathway.

Endocytosis is a critical regulatory mechanism for signaling pathways to fine-tune the pathways’ duration and recycling of pathway components (39). AMOT recycles tight junction proteins (i.e., Pals1/Patj, Par3) via its PDZ binding domain (9). The ACCH domain of AMOT targets the aforementioned tight junctions proteins to endosomes (34). Even though mutation does not affect the PDZ binding domain and ACCH motif in AMOT^ΔN^, congenital hydrocephalus in patients directed us to evaluate any change in barrier integrity and early endocytosis level of the AMOT/AMOT^ΔN^–expressing cells. We added BMP6 stimulation to the experimental setup and analyzed AMOT130^ΔN^- or AMOT130-positive endocytic vesicles. Quantification of AMOT in early endosomes demonstrated that overexpressing AMOT130^ΔN^ or AMOT130 elevated the number of AMOT-positive endocytic vesicles, in line with the previous observations (9). However, BMP6 stimulation elevated the number of AMOT130^ΔN^-positive vesicles when compared with AMOT130-positive vesicles. Because BMP6 stimulation increased the levels of AMOT130^ΔN^ in early endosomes, we speculated that endocytosis is a mechanism to fine-tune proper levels of AMOT at the cell surface.

Disrupted cell-cell junction integrity contributes to the pathology of hydrocephalus (40–42). With TEER and permeability assays, we demonstrated that polarized epithelial cells expressing the variant AMOT130^ΔN^ significantly decreased cell barrier integrity. Taking into account an angiogenesis-modulating function of AMOT and dilated brain vessels in AMOT knockout embryonic mice (10), we investigated the potential localization and cell-cell junction integrity change in wild-type or mutant AMOT-expressing hCMECs. Wild-type or mutant AMOT overexpression leads to apical and basal side localization of AMOT. TEER and permeability measurements indicated a destabilized barrier integrity in AMOT130-overexpressing and AMOT130^ΔN^-expressing hCMECs, again highlighting the importance of delicate regulation of AMOT130 degradation. By showing altered barrier integrity in AMOT130^ΔN^ expressing epithelial and endothelial cells, we indicated that the cells expressing AMOT130^ΔN^ could not hold the junctions tight enough, leading to leakiness that leads to the pathology of hydrocephalus.

In summary, the identified *AMOT* pathogenic variant is associated with congenital hydrocephalus, which becomes evident only in the third trimester of gestation. After early VP shunt insertion, the patients exhibit normal development, suggesting that the AMOT-related disease might be milder than that associated with *L1CAM*. This conclusion should be considered with caution because of the relatively short period of follow-up, the small number of patients in our cohort, and the unique pathomechanism associated with the p.Met1Thr (AMOT130^ΔN^) variant. For example, other *AMOT* disease-causing variants, associated with abnormally low AMOT protein levels, may manifest in a more severe phenotype. Nonetheless, the finding of pathogenic *AMOT* variants in families with X-linked hydrocephalus may still be used for preimplantation genetic diagnosis. By considering this newly identified mechanism, alternative and effective therapies against congenital hydrocephalus can be developed. Finally, the normal development of adult patients who have undergone early VP shunt insertion advocates for the preservation of ongoing pregnancies of AMOT-associated X-linked hydrocephalus.

## Methods

### Sex as biological variable.

Affected individuals of X-linked congenital hydrocephalus are male. Our studies compared male patient-derived skin fibroblasts with male and female donor-derived skin fibroblasts, and we indicated that our findings are specific to male patients.

### Genetic investigation.

After written informed consent was obtained, exome analysis was performed on DNA extracted from the blood of the proband IV1 (single exome; Figure 1). ES was performed with the IDT kit (xGen Exome Research Panel version 1.0) on the NOVASEQ 6000. Exonic sequences from genomic DNA were enriched with the SureSelect Human All Exon 50 Mb V5 Kit (Agilent Technologies). Sequences were generated on a HiSeq2500 sequencing system (Illumina) with 125 bp paired-end runs. Read alignment and variant calling were performed with DNAnexus using default parameters with the human genome assembly hg19 (GRCh37) as the reference. Filtering was performed as described elsewhere (43). Exome analysis of the proband yielded 53 million reads, with a mean coverage of 78×, and 94% over 20×.

### Segregation studies.

An amplicon containing the *AMOT* variant was amplified by conventional PCR of genomic DNA from probands and all available family members and analyzed by Sanger dideoxy nucleotide sequencing.

### Cell culture.

Human skin fibroblasts (HSFs) from proband IV (a male patient), a male individual acting as a control, and a female individual acting as a control were isolated from skin-punch biopsies and expanded in RPMI (Lonza) supplemented with 20% FCS, 2 mM l-glutamine (l-Glu), and 1% penicillin (100 U/mL) plus streptomycin (0.1 mg/mL) (P/S). MCF7 and HEK293T cells were grown in DMEM (PAN Biotech) supplemented with 10% FCS, 2 mM l-Glu, and 1% P/S. We cultured hCMECs/D3 (Cedarlane, CELLutions Biosystems) in endothelial growth medium (EGM2) with supplements (Lonza; CC-3162, without heparin). BMP6-stimulation experiments were performed with starvation medium (without FCS supplement to cells respective growth medium containing 2 mM l-Glu and 1% P/S) by starving HSFs for 3 hours and MCF7 and HEK293T cells for 5 hours. BMP6 (from S. Vukicevic, University of Zagreb, Croatia) final concentration during stimulation for the different cell lines was 10 nM.

### Generation of overexpression plasmids.

Site-directed mutagenesis was performed to generate human AMOT130 and AMOT130^ΔN^ plasmid DNAs. FLAG-tag was removed from previously generated AMOT130 plasmid (11). First, the 91 amino acids from the beginning of N-terminus and, later, the FLAG tag were deleted from AMOT130-FLAG plasmid DNA to generate AMOT130^ΔN^ plasmid DNA. Chimeric proteins were generated by fusing the first 91 amino acids of AMOT130 (C1), the first 109 amino acids of AMOT130 (includes LPTY between amino acids 106 and 109) (C2) to the N-terminus of AMOT80. C3 plasmid was generated by mutating the LPTY region to AATY in the C2 plasmid. The second amino acid after methionine, glutamic acid (E), of AMOT130^ΔN^ was mutated to arginine (R) to obtain E2R_AMOT130^ΔN^ plasmid. The TBD_AMOT130^ΔN^ construct was generated by adding the sequence of wild-type AMOT130, encoding for amino acids 73–91, which includes TBD^77–84^ (amino acids RQEPQG) to the N-terminus of AMOT130^ΔN^. The HA-BMPR2 plasmid was subcloned from the previously described pcDNA1 plasmid (44) to pcDNA3.1. Before conducting experiments, each plasmid DNA sequence was confirmed by DNA sequencing (Microsynth AG).

### Cell transfection.

MCF7 cells were seeded at the density of 2 × 10^–5^/well in 12-well plates for (a) AMOT130, AMOT130^ΔN^, and chimeric protein overexpression; (b) XAV-939 (10 μM; Selleckchem), JW 55, and WIKI4 (10 μM; MedChemExpress) treatment; (c) BMP6 stimulation; and (d) IF staining (cells seeded on coverslips) experiments. For apical-basal BMP6 stimulation, 5 × 10^–6^ cells/well were seeded in 6-well plates. One day after seeding, cells were transfected with the indicated plasmids with Lipofectamine 2000 (Invitrogen) following modified manufacturer’s instructions. Transfection mix prepared in Opti-MEM was added to cells for 5 hours. During transfection, cells were cultured in 2% FCS containing antibiotic-free DMEM medium. The transfection mix containing medium was replaced with full medium after 4 hours. One day after transfection, cells were transferred to Transwell plates and cultured for 2 days before apical/basal stimulation. For the TNKS inhibitor experiments, inhibitors (10 μM) were added for 12 hours 1 day after transfection. Other mentioned experiments were carried out 2 days after transfection. Before lysis, cells were cultured in the starvation medium for chimeric protein and XAV-939 inhibitor addition (3 hours for HSF) experiments.

TEER and permeability assays were carried out with wild-type– and AMOT130^ΔN^ expression–enriched MCF7 cells. Enrichment of the AMOT130^ΔN^-expressing MCF7 cells was done as follows. MCF7 cells, cultured in a T75 cell culture flask, were transfected using with Lipofectamine 2000 with the aforementioned AMOT130^ΔN^-overexpression plasmid DNA, which was linearized with EcoRV. Two days after transfection, cells were cultured in G418 (1.2 mg/mL) containing full medium and, every 2 days, medium was refreshed. After 2 weeks of selection, cells were seeded to Transwell plates. AMOT130^ΔN^-expressing and wild-type MCF7 cells were cultured in phenol red-free DMEM supplemented with 10% FCS, 2 mM l-Glu, and 1% P/S medium for 6 days for the TEER and permeability assays.

Transfection of hCMECs with EcoRV enzyme-restricted AMOT130- or AMOT130^ΔN^-overexpression plasmid DNAs was performed using Lipofectamine 2000 on the same day the cells were seeded for IF, TEER, and permeability assays. Two days after transfection, the medium was changed to EGM2 medium with supplements containing G418 (1 mg/mL). Every 2 days, G418-containing medium was refreshed. hCMECs were kept in the culture with 0.5 mg/mL G418 EGM2 medium with supplements until the IF, TEER, and permeability assays.

HSF cells were seeded at the density of 6 × 10^–4^/well in 24-well plates for UBR4 knockdown. Accell targeting UBR4 and nontargeting (scr) siRNA were purchased from Dharmacon Reagents and 80 nM was decided for their final concentrations. Lipofectamine 2000 was used as a transfection agent. During transfection, cells were cultured in Opti-MEM. A transfection mix prepared in Opti-MEM was added to cells, and medium was changed to full medium after 5 hours. After cells were incubated in full medium for 48 hours, they were incubated in starvation medium for 3 hours and lysed for immunoblotting.

For CO-IP experiments, HEK293T cells were seeded in 10 cm dishes at a density of 4 × 10^–6^ cells/dish. The next day, each dish was transfected with the respective plasmid DNA constructs: AMOT130 and HA-BMPR2, AMOT130^ΔN^ and HA-BMPR2, pcDNA3.1. For biotinylation experiments, 1 × 10^–6^ HEK293T cells were seeded in 6 cm dishes and transfected with the indicated plasmid DNAs by using PEI (Sigma-Aldrich) according to manufacturer’s instructions.

### Immunoblot analysis.

Protein lysate separation was performed with SDS-PAGE. Subsequently, proteins were transferred on PVDF membranes. Membranes were blocked for 1 hour with 3% w/v bovine serum albumin in TBS-T. Afterward, they were incubated with respective primary antibodies overnight in the cold room (4°C). Membranes were incubated with HRP-conjugated secondary antibodies corresponding to primary antibodies for 1 hour at room temperature. Chemiluminescent reactions were carried out with WesternBright Quantum HRP substrate (Advansta) and imaged by Fusion-FX7 detection system (Vilber-Lourmat). Primary antibodies’ suppliers and their dilutions were as follows: AMOT (Bethyl Laboratories, A303-305A; 1:1,000); phosphorylated SMAD1/5 (Cell Signaling Technologies, clone 41D10; 1:2,000); GAPDH (Cell Signaling Technologies, clone 14C10; 1:8,000); HA tag antibody (Sigma-Aldrich, clone HA7; 1:1,000); and UBR4 (p600) (Bethyl Laboratories, A302-278A; 1:2,000). Western blot images were quantified by ImageJ (Fiji-win64).

### Quantitative real-time PCR analysis.

RNA was isolated with the NucleoSpin RNA XS isolation kit (Macherey-Nagel), following manufacturer’s instructions. Total RNA (0.5–1 μg) was subjected to reverse transcription by using random primers (100 pmol/μL; Invitrogen) and M-MuLV reverse transcriptase enzyme (200,000 U/μL; New England Biolabs). Real-time PCR was performed using the StepOne Plus Real-Time PCR System (Thermo Fisher Scientific) with specific primers as follows: forward AMOT130 primer 5′ TGAAGAAGCCAAGGTCCAGT 3′, reverse AMOT130 primer 5′ CCCTCATCTTGGTGCATCTT 3′; and forward RSP9 primer 5′ CTGCTGACGCTTGATGAGAA 3′, reverse RSP9 primer 5′ CAGCTTCATCTTGCCCTCAT 3′. Luna PCR MASTER Mix (New England Biolabs) was used doing triplicate reactions in MicroAmp Optical 96-well reaction plates (Thermo Fisher Scientific). Fold induction was calculated using the comparative Ct method by analyzing relative AMOT130 expression to the housekeeping gene RSP9.

### CO-IP studies.

Two days after transfection, cells were starved for 5 hours and stimulated with BMP6 (10 nM) for 30 minutes. Cell were lysed and scraped after adding RIPA lysis buffer (150 mM NaCl, 25 mM Tris/HCl, 0.1% SDS, 0.5% NP-40 [pH 7.8]) supplemented with inhibitors (1 mM PMSF, 2 mM Na_3_VO_4_, 20 mM Na_4_P_2_O_7_, 50 mM NaF, complete protease inhibitor cocktails [Roche]). Cell debris were removed via centrifugation. Total cell lysates were collected from each sample, and the supernatants were incubated with anti-HA (1 μg/mL) or isotype control (IgG control) antibodies on a wheel rotator at 4°C overnight. Protein A-Sepharose beads (GE Healthcare) were added to precipitate immunocomplexes at 4°C for 2 hours. Beads were washed 5 times with inhibitor containing lysis buffer. Laemmli sample buffer (2×) was added to samples and boiled for 5 minutes at 95°C. Then samples were subjected to Western blotting.

### Surface biotinylation assays.

Two days after transfection, cells were subjected to 5 hours of starvation and stimulated with BMP6 (10 nM) for 30 minutes. Proteins at the cell surface were biotinylated with cell-impermeable 0.5 mg/mL EZ-Link Sulfo-NHS-SS-Biotin (Thermo Fisher Scientific) dissolved in PBS+ MgCl_2_ (10 mM) at 4°C for 50 minutes. As a control to biotinylation, control samples were incubated with PBS-MgCl_2_ (10 mM) for 50 minutes. Quenching was performed with 50 mM Tris, pH 8.0. Cells were lysed with RIPA lysis buffer with freshly added inhibitors (the same as used for CO-IP). After scraping, samples cell debris was removed by centrifuging. Supernatants of samples were collected for total cell lysate. The rest of the supernatants were incubated with streptavidin-coupled Sepharose beads (GE Healthcare) on a wheel rotator at 4°C overnight. The beads were washed 5 times with RIPA lysis buffer supplemented with inhibitors. Samples were eluted with 2× Laemmli sample buffer and subjected to Western blotting.

### IF microscopy and quantification of the staining.

Two days after transfection, MCF7 cells were cultured in starvation medium for 5 hours, fixed with 4% PFA, quenched with 50 mM ammonium chloride, and permeabilized with 0.3% Triton-X in PBS. The same procedure was applied to hCMECs selected for AMOT130 or AMOT130^ΔN^ overexpression: cells were cultured for 4 days in EGM2 medium with supplements. Blocking solution, 10% normal goat serum in 2% bovine serum albumin, was added to cells for 1 hour at room temperature. The respective primary antibodies were prepared in blocking solution and incubated at 4°C overnight. Primary antibodies and their dilutions were AMOT (Bethyl laboratories, A303-305A; 1:300) and EEA1 (BD Transduction Laboratories, 610456; 1:200). The next day, cells on coverslips were incubated with anti-rabbit Alexa Fluor 488 (Invitrogen, A11034; 1:500 dilution) and anti-mouse Alexa Fluor 594–conjugated antibodies (Invitrogen, A11005; 1:300 dilution) for 1 hour at room temperature. Nuclei were stained with DAPI. Imaging was carried out with an inverted confocal SP8 microscope (Leica). The same procedure was applied to stain AMOT and PKC-ζ (Santa Cruz, sc-17781; 1:200) proteins in HSF cells.

Quantification of apical-basal AMOT localization was performed using the orthogonal sections of images of each condition from the 3 independent experiments. AMOT-overexpressing cells were identified, and Z-stack images were exported via LAS X software. Apical and basal sides were separated and masked manually using GIMP 2.10.32 image editing software. After image processing, the images were analyzed via a self-written Python script (https://github.com/agknaus/Hastar-et-al.-2024-Image-analysis; commit ID 101fb84). The program identified and determined the percentage of the fluorescent signal on the green channel by examining the red-green-blue color code of each pixel.

### Quantification of EEA1–AMOT130/AMOT130^ΔN^ colocalization staining.

Quantification of Z-stacks of confocal images stained with EEA1 and AMOT130/AMOT130^ΔN^/pcDNA3.1 was performed in ImageJ using a self-written script (https://github.com/agknaus/Hastar-et-al.-2024-Image-analysis; commit ID 790a0f5). Within the confocal images, EEA1-rich vesicles were identified via the AnalyzeParticles function after background subtraction in regions defining the cells. These particles were translated into masks, and the AMOT intensity in each vesicle was measured. Furthermore, the mean AMOT intensity in all images within cellular regions was measured. In Python, the EEAs were marked as AMOT130/AMOT130^ΔN^-positive if the AMOT intensity was higher than the mean intensity of the respective AMOT variant within the cell. For the exact parameters used, refer to the scripts.

### TEER and permeability assays.

Wild-type or AMOT130^ΔN^-overexpressing MCF7 cells were seeded into 6.5 mm Transwell 0.4 μm inserts (Corning) at the density of 1.5 × 10^–^5 and cultured for 6 days until cells reached confluency. Similarly, wild-type, AMOT130-, or AMOT130^ΔN^-overexpressing hCMECs (1.5 × 10^–5^ per well) were also cultured in 6.5 mm Transwell 0.4 μm inserts for 4 days. The medium of the cells was changed daily. Resistance of the barriers were measured with Trans-Filter Adaptor of Electric Cell-Substrate Impedance Sensing device. Data were recorded in multiple frequencies for at least 2 hours. Data obtained at 62.5 Hz frequency were plotted in the graphs. After completing TEER, 4 kDa FITC-dextran (1 mg/mL; Sigma-Aldrich, 46944) containing phenol–red-free DMEM supplemented with 10% FCS, 2 mM l-Glu, and 1% P/S (phenol red-free full medium) were added to the upper compartments of the Transwell plates containing MCF7 cells. FITC-dextran (70 kDa; 1 mg/mL; Sigma-Aldrich, 46945) dissolved in EGM2 with supplements was added to the upper chambers of the Transwell plates for hCMECs. The basal part of the wells was filled with phenol red-free full medium and EGM2 medium with supplements for MCF7 and hCMECs, respectively. After 4 hours, the basal medium was collected from Transwell plates. Fluorescence intensity was measured with an Tecan Infinite F200 PRO microplate reader with 485 emission and 535 excitation wavelengths.

### Figures.

Figure schemes were generated with BioRender.com (https://BioRender.com/tw5ah2z).

### Statistics.

Statistical tests were carried out with GraphPad Prism software. Respective statistical tests are listed in the figure legends. Unpaired 2-tailed *t* test, ordinary 1-way ANOVA with Tukey’s or Dunnett’s multiple comparison post hoc tests, or 2-way ANOVA with Tukey’s or Šídák’s multiple comparisons tests were used depending on the experiment. An α level of less than 0.05 was considered statistically significant in all experiments.

### Study approval.

Written informed consent from the participants to publish the MRI images and results with the HSF cells was obtained prior to their participation in this study, according to the IRB-approved protocol 0306-10-HMO from the Hadassah Medical Center (Jerusalem, Israel).

### Data availability.

ES data are uploaded to the ClinVar database (accession SCV006076459). Code for IF microscopy quantification is available on GitHub (https://github.com/agknaus/Hastar-et-al.-2024-Image-analysis; commit ID 101fb84 & aff6cec). The values of all data points in graphs are reported in the Supporting data values file.

## Author contributions

HD and OE identified the patients with AMOT mutation and supplied clinical information and MRI images, and performed a genetic investigation. OE established isolation of the patient fibroblasts and provided control fibroblasts. NH, NKT, and PK planned the experimental strategies. NH and NKT planned and generated overexpression plasmids, quantified the Western blot images, and performed CO-IP and biotinylation assay. NKT designed and drew graphical schemes in figures; performed overexpression and AMOT levels analysis, TNKS inhibition, BMP6 stimulation, and IF staining in MCF7 cells; and quantified the apical-basal AMOT level in IF staining. NH performed AMOT expression analysis, TNKS inhibition, BMP6 stimulation, and IF staining in HSF cells; performed imaging of all IF staining; and conducted TEER and permeability assays in MCF7 cells and hCMECs. LO quantified the EEA1-AMOT colocalization staining in MCF7 cells. PV provided the hCMECs cell line. PK supervised the study and provided funding. NH, NKT, HD, GG, OE, and PK wrote the manuscript.

## Supplementary Material

Supplemental data

Unedited blot and gel images

Supporting data values

## Version 1 09/02/2025

Electronic publication
