# Supplementary material for: A pathogenic variant of AMOT leads to isolated X-linked congenital hydrocephalus due to N-terminal truncation
Source: J Clin Invest. 2025 Sep 2;135(17):e179438. doi: 10.1172/JCI179438 (PMC12404752; doi:10.1172/JCI179438)
Supplement: Supplemental data [file jci-135-179438-s067.pdf]

A B

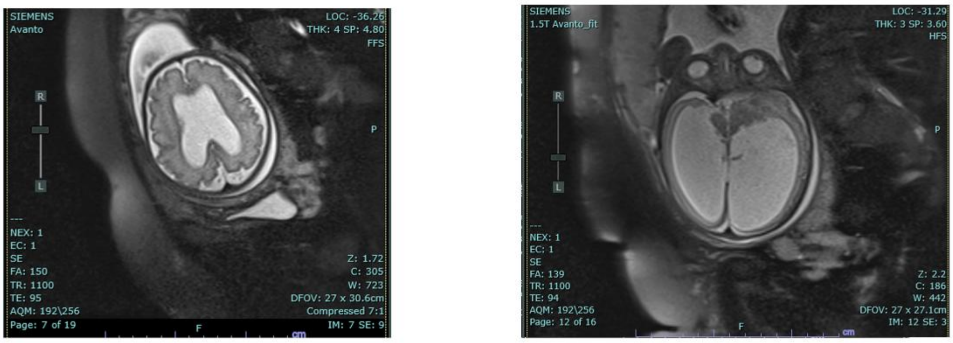

Fetal brain MRI of proband IV6,
26+1 weeks
(prior to termination of pregnancy)

Fetal brain MRI of proband IV7

Supplemental Figure 1. **Fetal brain MRI images. (A)** Brain MRI of fetus IV6 at week 27 demonstrating enlargement of ventricles. **(B)** Brain MRI of fetus IV7 at week 32 disclosing compressed brain parenchyma due to hydrocephalus.

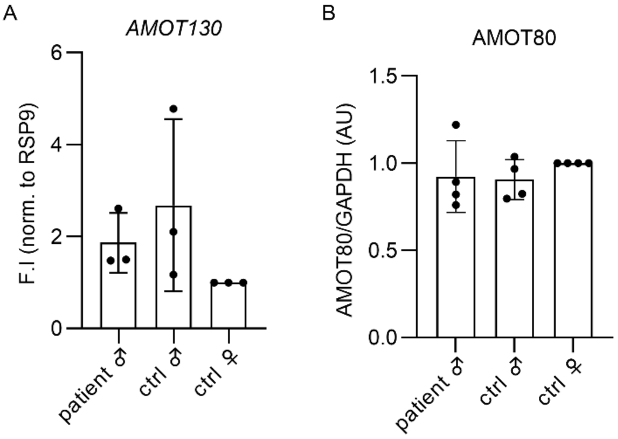

Supplemental Figure 2. Mutation does not change AMOT130 mRNA and AMOT80 protein levels. (A) AMOT130 gene expression level in primary human skin fibroblast cells isolated from proband IV (patient ♂), male (control ♂), and female control (control ♀). **(B)** Quantification of AMOT80 protein level in male patient (patient ♂), male control (control ♂), and female control (control ♀) primary skin fibroblast cells from western blot analysis.

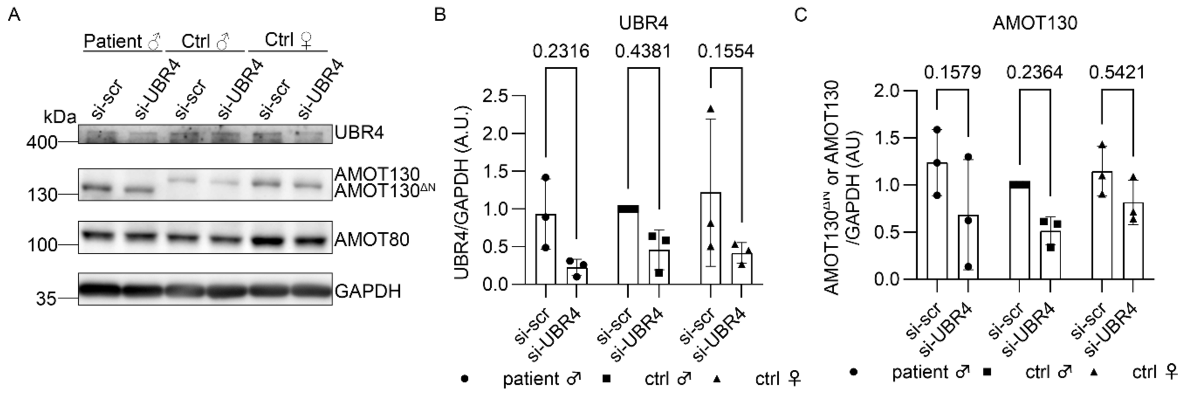

D

Gene	Protein Accession #	Protein Description	Log2Folds siRNA2	p-value	SIG S2
Ubr4	sp|A2AN08|UBR4_MOUSE	E3 ubiquitin-protein ligase UBR4 OS=Mus musculus GN=Ubr4 PE=1 SV=1	-1.38	6.99E-08	TRUE
Nedd4	sp|P46935|NEDD4_MOUSE	E3 ubiquitin-protein ligase NEDD4 OS=Mus musculus GN=Nedd4 PE=1 SV=3	0.84	5.69E-05	TRUE
Nedd4-2	sp|Q8CFI0|NED4L_MOUSE	E3 ubiquitin-protein ligase NEDD4-like OS=Mus musculus GN=Nedd4l PE=1 SV=2	0.43	0.001	

E

Gene	Log2Folds siRNA2	p-value	SIG S2
Ubr4	-1.85	0.001	TRUE
Nedd4	0.31	0.043	FALSE
Nedd4-2	0.07	0.81	FALSE

Supplemental Figure 3. UBR4 knockdown tends to decrease wild-type and mutant AMOT130 protein levels. **(A)** Representative western blot showing UBR4, AMOT130/ AMOT130$^{\Delta N}$, AMOT80, and GAPDH protein levels after siUBR4 knockdown or scr-siRNA application to primary skin fibroblasts. Quantification of **(B)** UBR4, **(C)** AMOT130$^{\Delta N}$, and AMOT130 protein levels from 3 independent experiments in si-scr and si-UBR4 treated fibroblasts. P values are presented in the graphs and calculated by two-way ANOVA with Šídák's multiple comparisons tests. **(D)** Tandem mass tag mass spectroscopy and **(E)** RNA sequencing results of selected genes; Ubr4, Nedd4, and Nedd4-2 after UBR4 knockdown in the tibialis anterior muscle of mice. Tables are modified from Hunt et al. 2019.

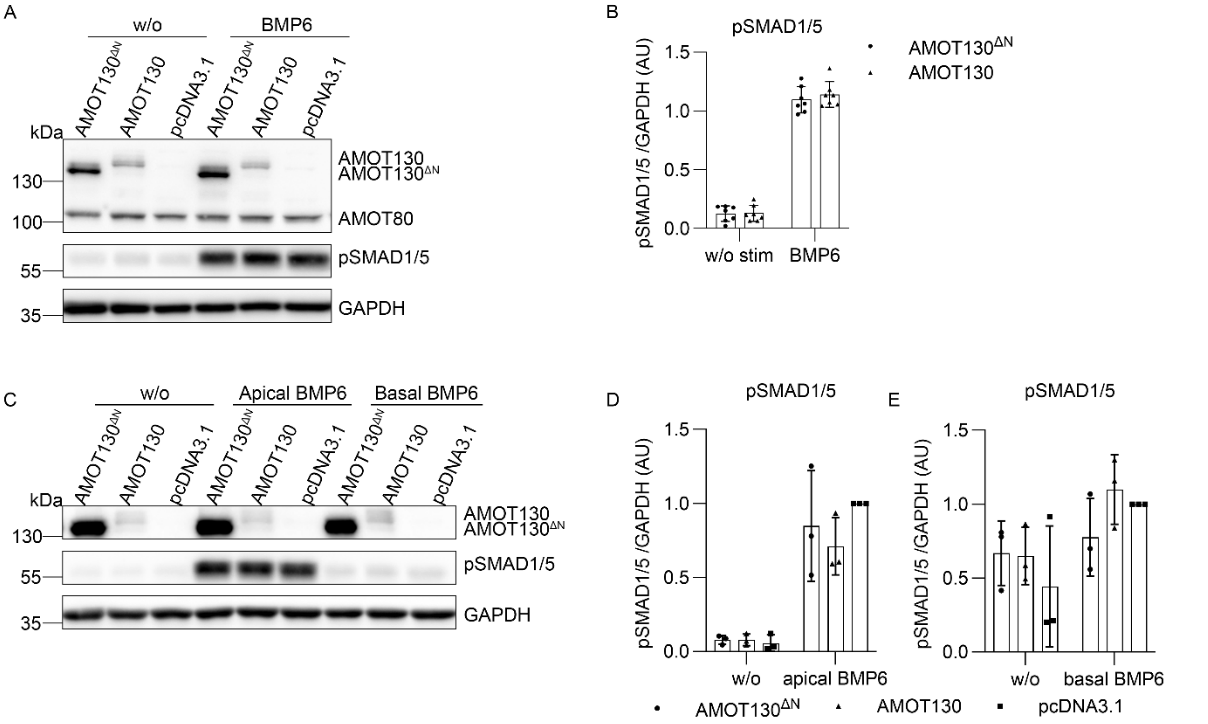

Supplemental Figure 4. SMAD1/5 phosphorylation is not affected in AMOT130$^{\Delta N}$ or AMOT130-expressing MCF7 cells. (A) Representative western blot depicting respective protein levels in AMOT130$^{\Delta N}$ or AMOT130-expressing MCF7 cells following 30 min BMP6 (10 nM) stimulation. **(B)** Quantification of pSMAD1/5 protein level from 7 independent experiments in AMOT130$^{\Delta N}$ and AMOT130-expressing MCF7 cells. **(C)** Western blot showing SMAD1/5 phosphorylation levels from apical or basal side BMP6 (10 nM) stimulated MCF7 cells expressing either AMOT130$^{\Delta N}$ or AMOT130; control transfection with pcDNA3.1. Quantification of pSMAD1/5 levels upon **(D)** apical and **(E)** basal BMP6 (10 nM) stimulation from 3 independent experiments.

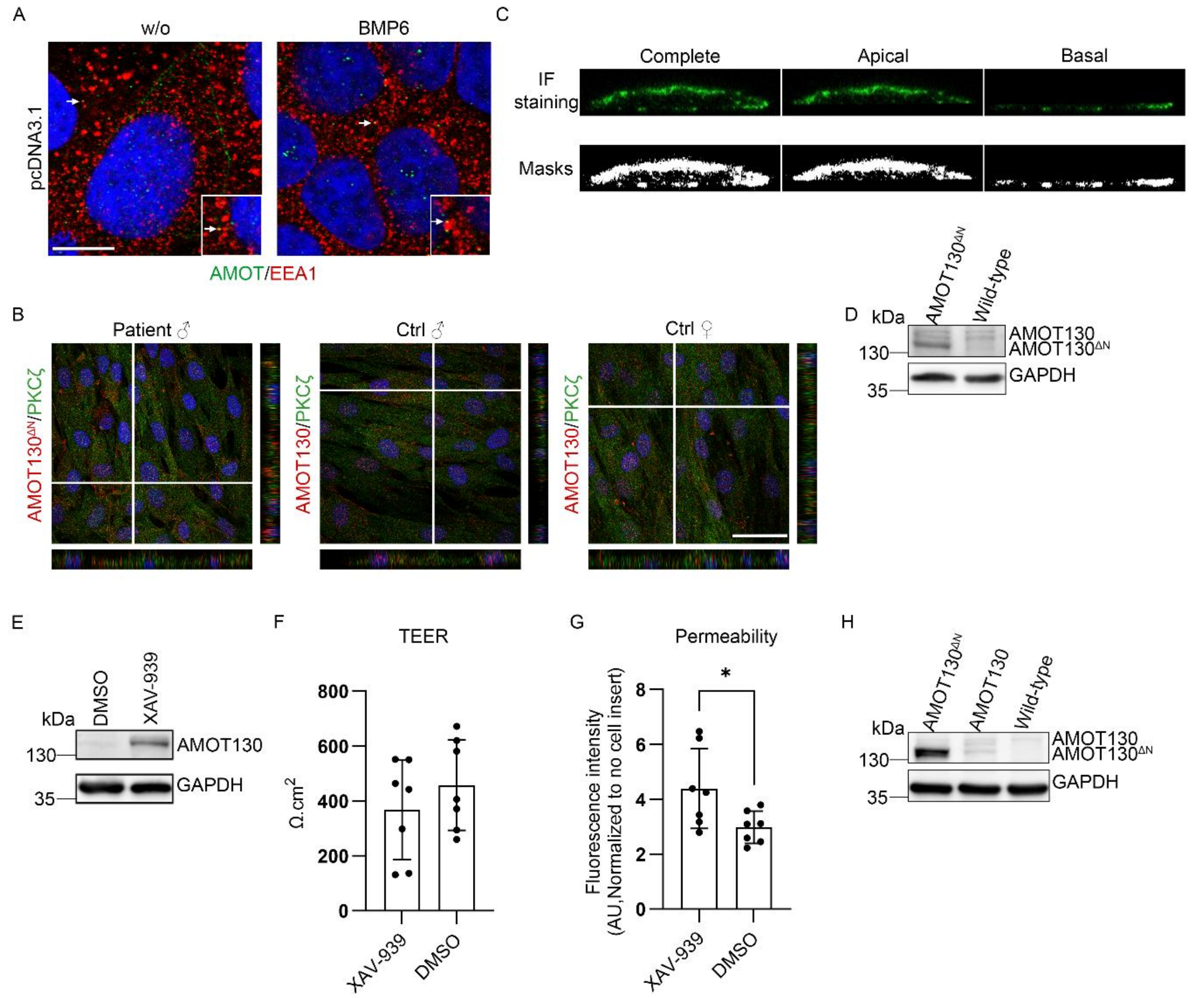

Supplemental Figure 5. (A) IF imaging results illustrating AMOT (green) and EEA1 (red) colocalization in yellow colors, as shown by arrows in zoom-ins in pcDNA3.1(transfection control) transfected MCF7 cells. (Scale bar = 10 µm) **(B)** AMOT (red) and PKC-ζ (green) co-staining in patient ♂, control ♂, and control ♀ fibroblasts. White lines indicate xz (lower) and yz (right) cross sections. (Scale bar = 50 µm) **(C)** Figure 6A AMOT130 xz section image to explain how the masking was performed to quantify apical vs. basal AMOT level quantification. The cell showing the full cell body (upper left), the apical side with manually masked basal side (upper middle), and the basal side with manually masked apical side (upper right). Representative images of the same cell showing the mask of the full cell body (lower left), the apical side (lower middle), and the basal side (lower right) were automatically generated by the analysis program. **(D)** Representative western blot indicating AMOT130$^{\Delta N}$ or AMOT130 expression in MCF7 after TEER and permeability assay. **(E)** A representative Western blot shows increased levels of AMOT130 following treatment with XAV-939, compared to the DMSO control after the permeability assay in MCF7 cells. **(F)** TEER measurement and **(G)** permeability assay results with MCF7 cells cultured for 4 days and treated with XAV-939 (10 µM) or DMSO for 2 days. * $P \leq 0.05$, unpaired t-test. **(H)** Western blot results depict AMOT130$^{\Delta N}$ and AMOT130 after the permeability assay in hCMECs.
