# Supplementary material for: A pathogenic variant of AMOT leads to isolated X-linked congenital hydrocephalus due to N-terminal truncation
Source: J Clin Invest. 2025 Sep 2;135(17):e179438. doi: 10.1172/JCI179438 (PMC12404752; doi:10.1172/JCI179438)

Full unedited blot for Figure 2 B

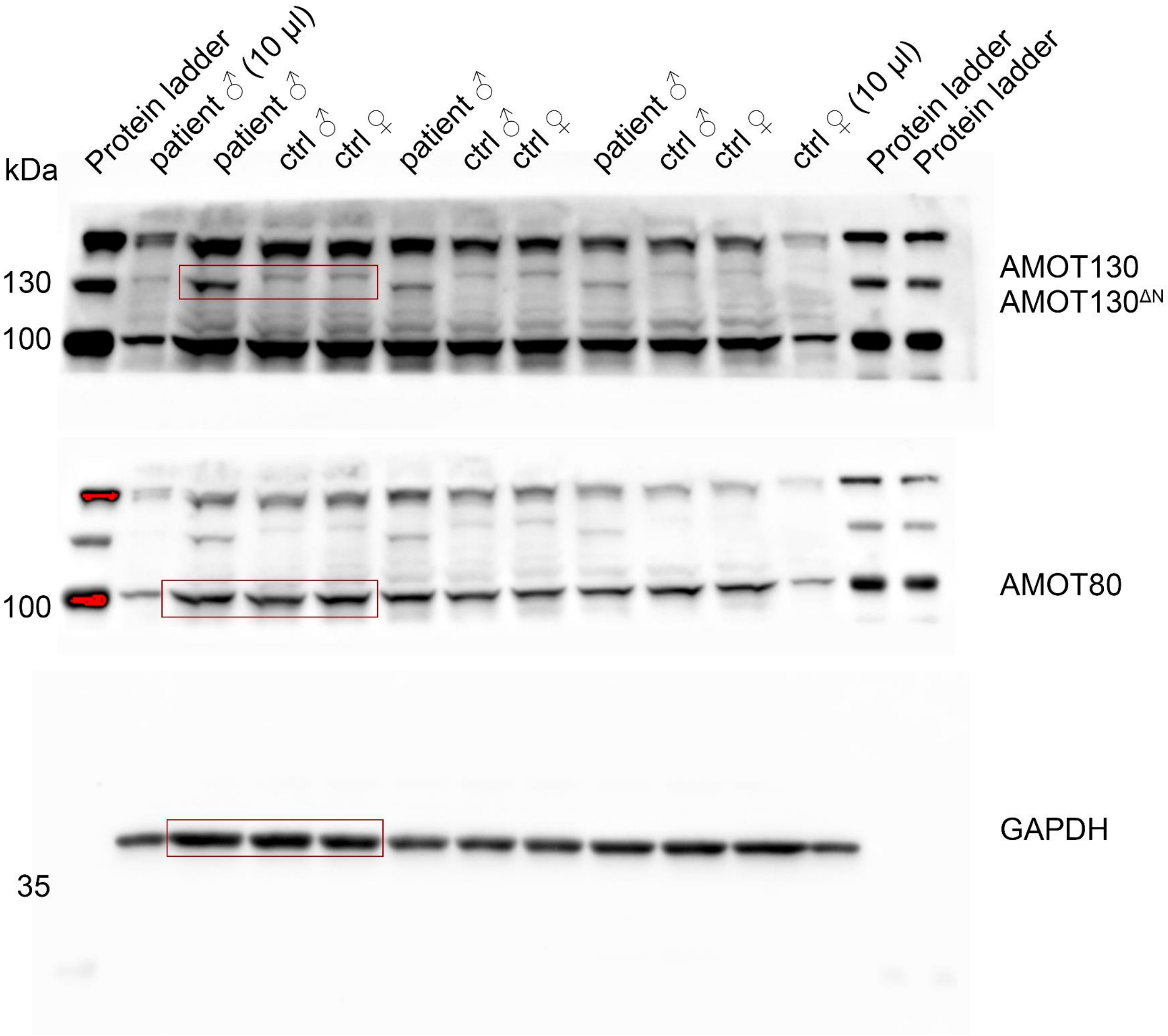

Full unedited blot for Figure 2 D

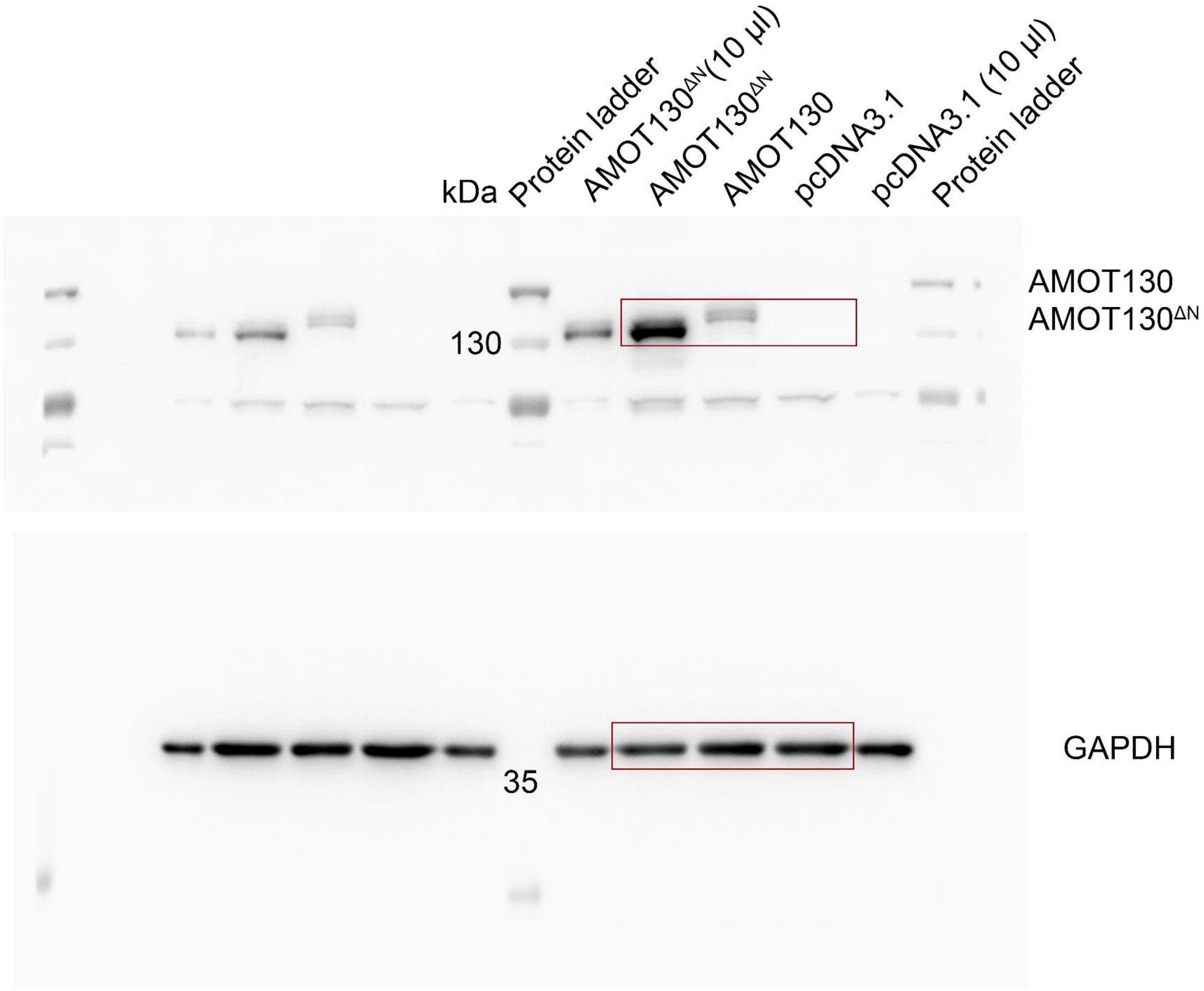

Full unedited blot for Figure 2 G

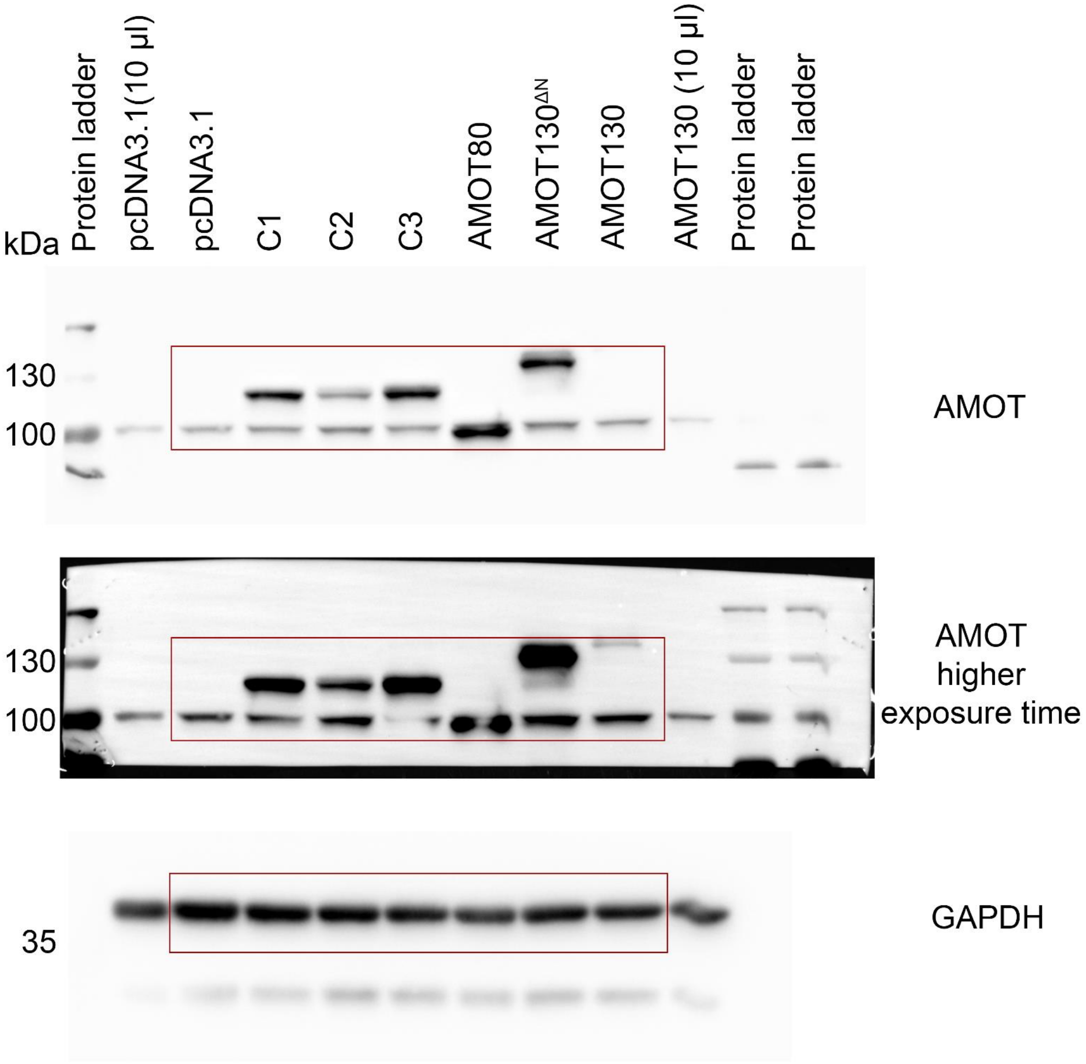

Full unedited blot for Figure 3 B

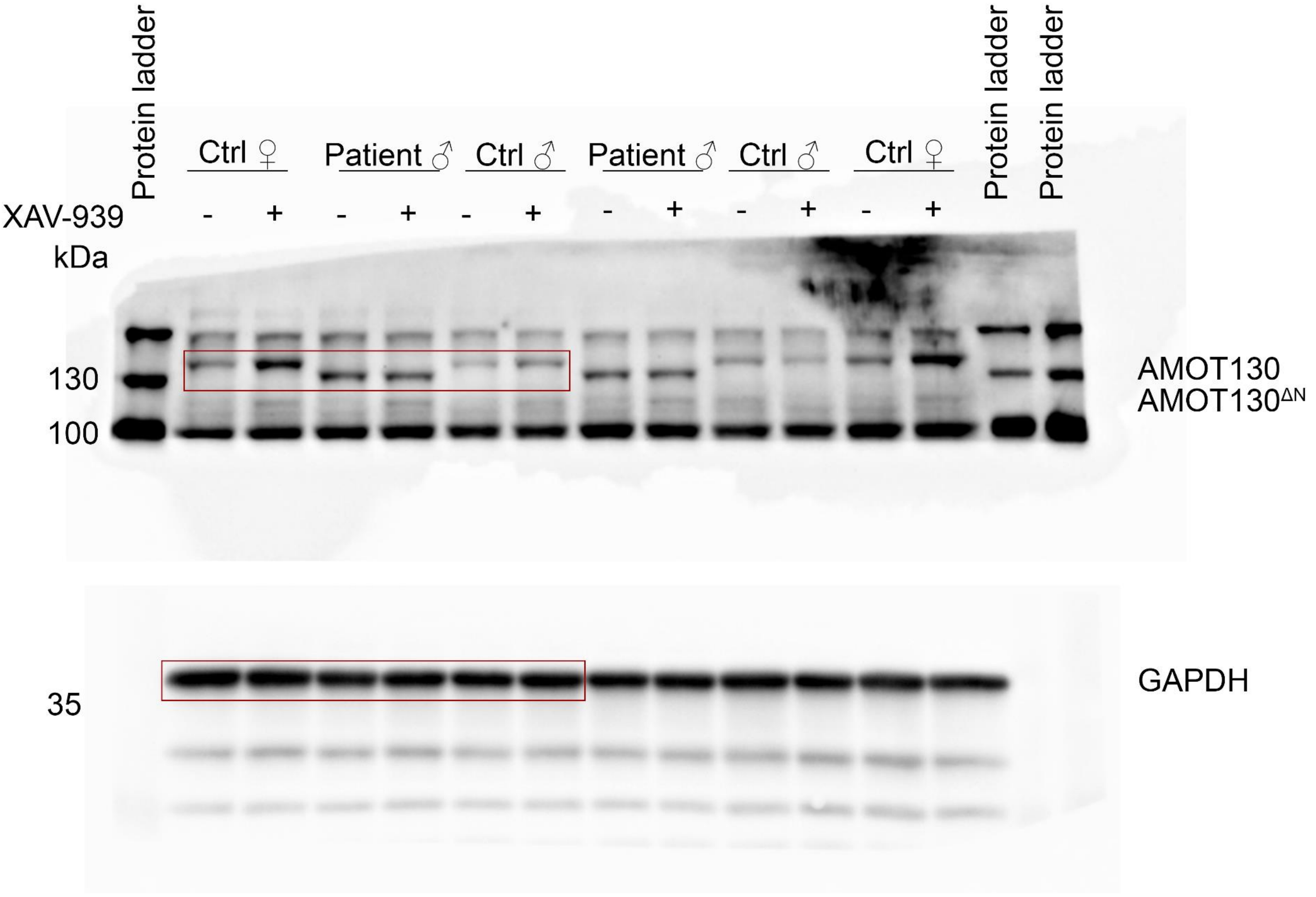

Full unedited blot for Figure 3 D

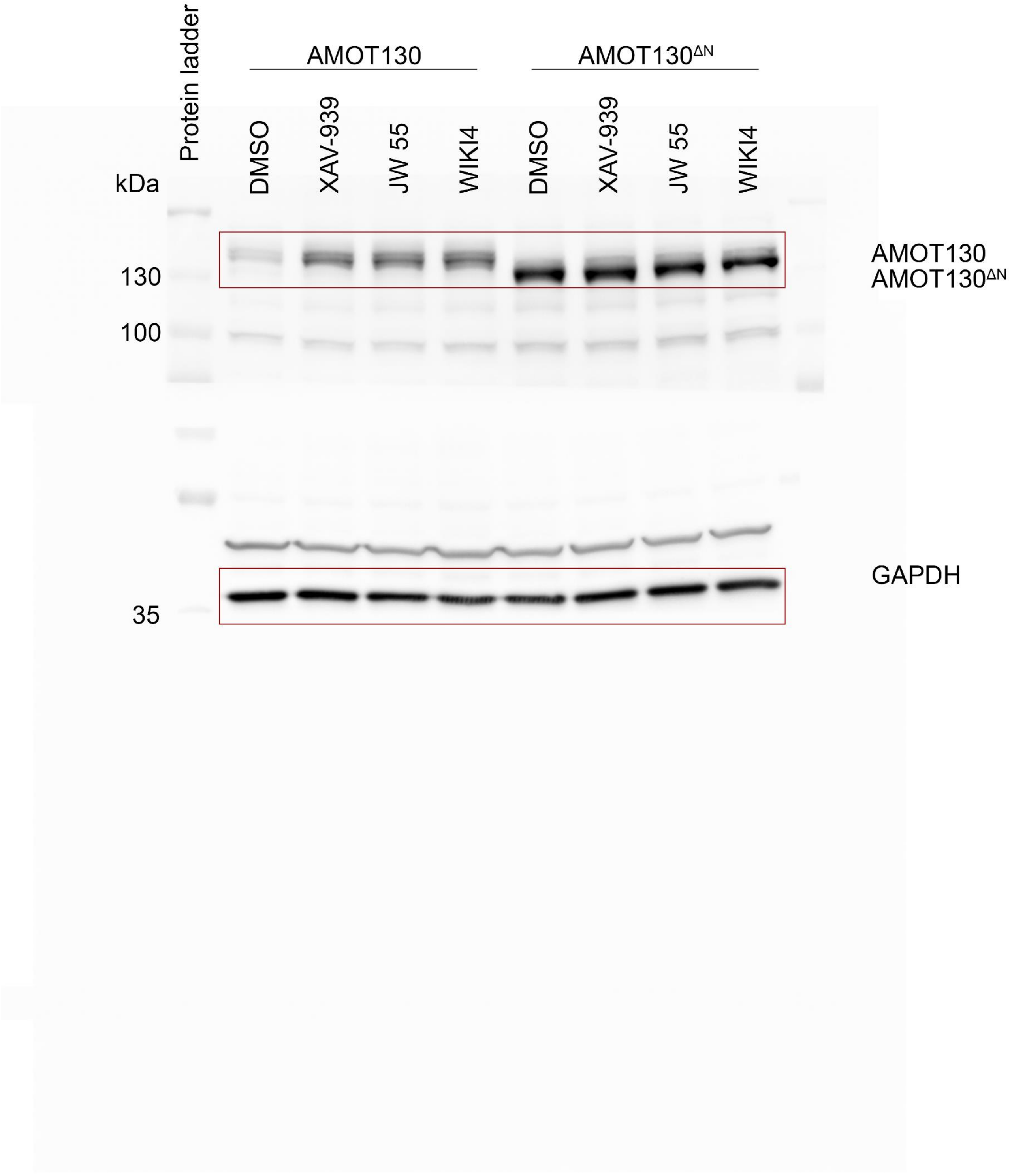

Full unedited blot for Figure 3 F

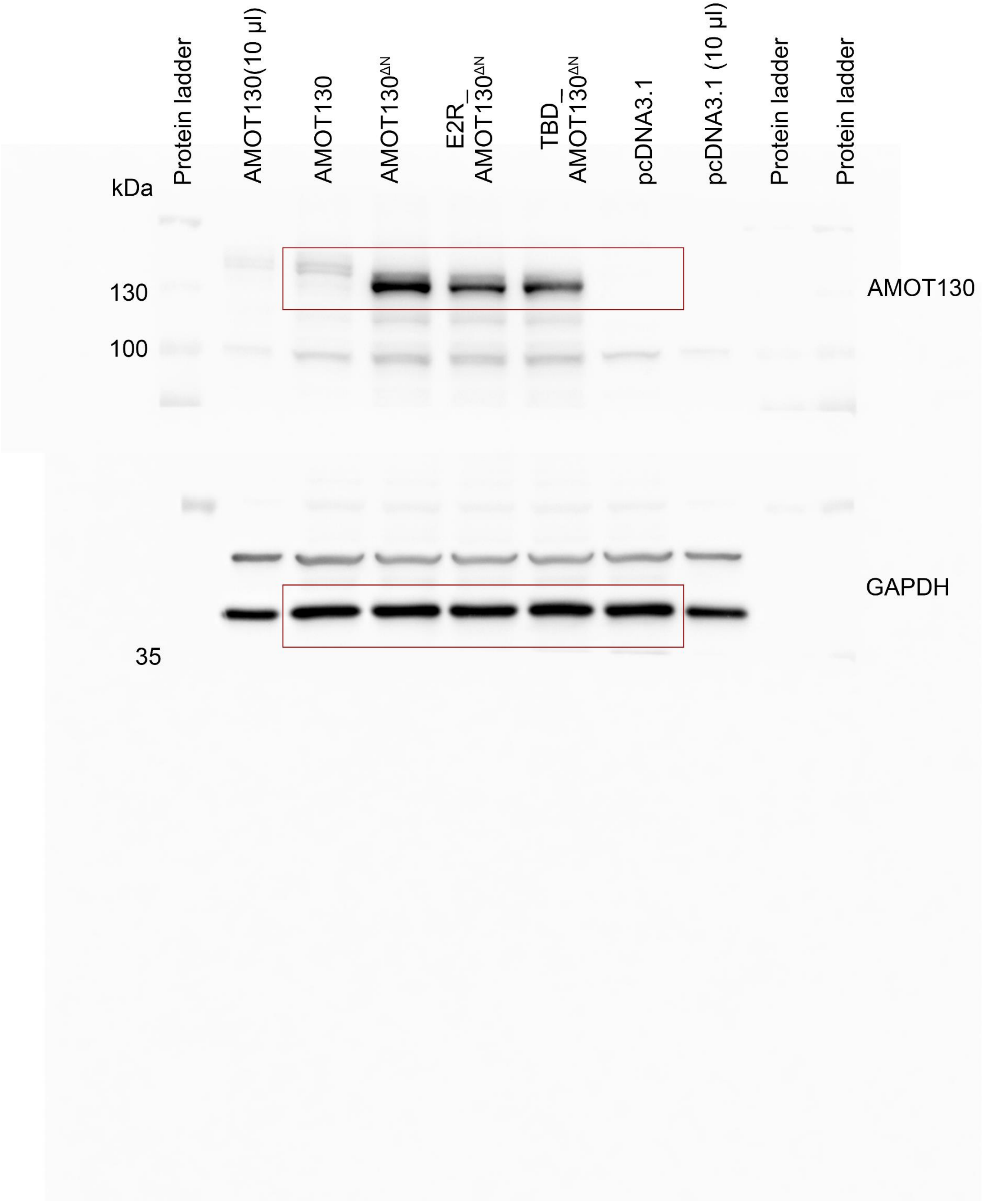

Full unedited blot for Figure 4 A

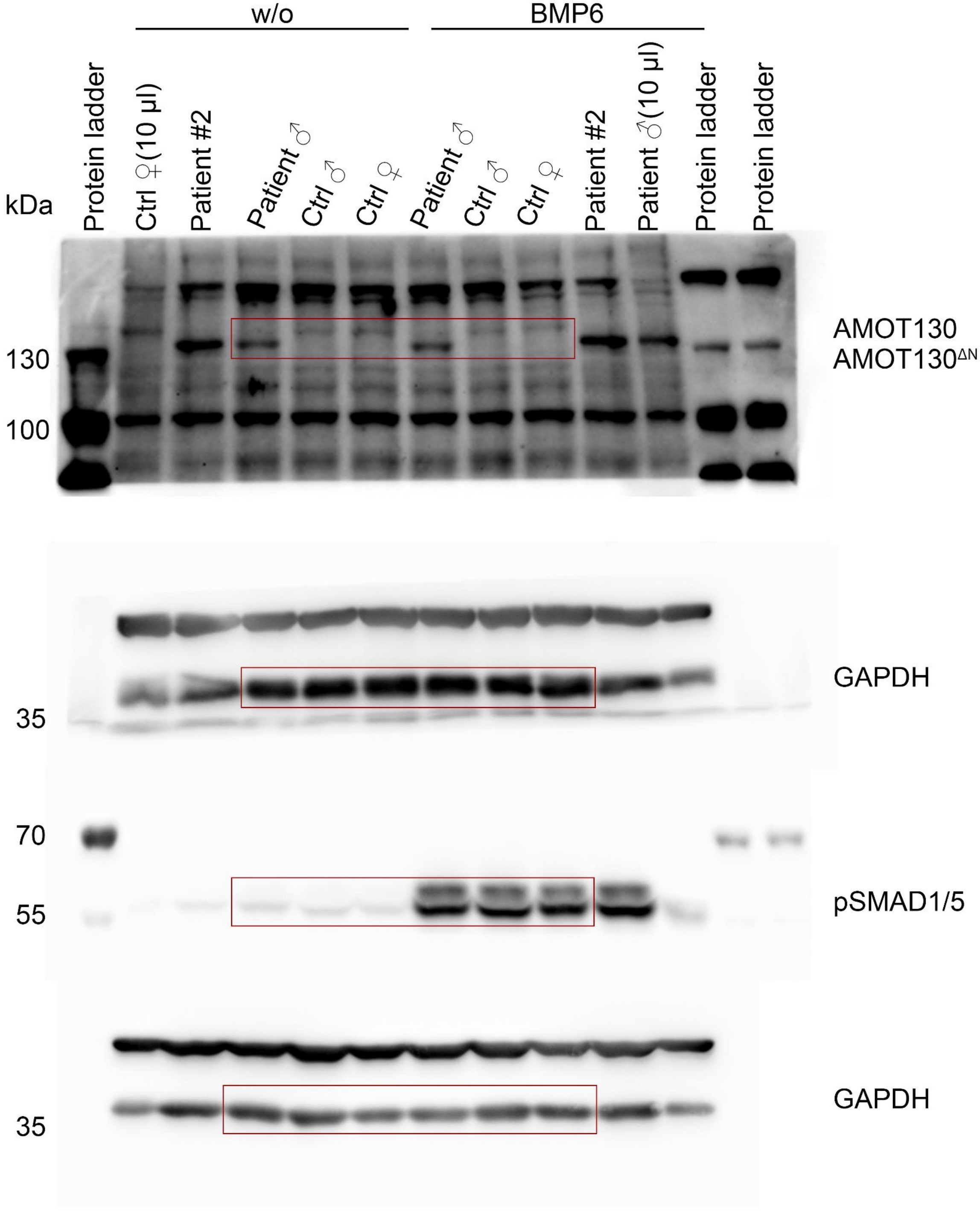

Full unedited blot for Figure 4 C_IP

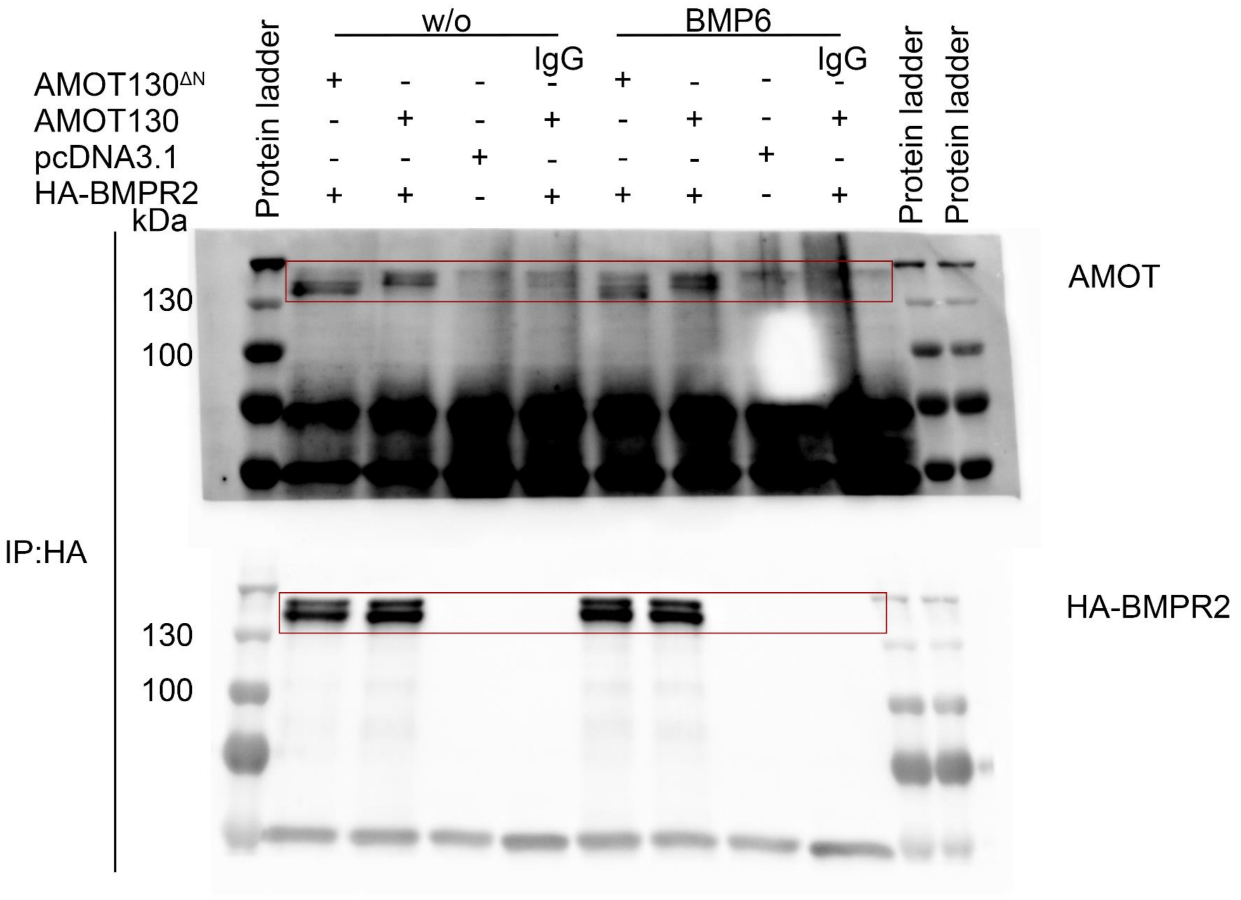

Full unedited blot for Figure 4 C_TCL

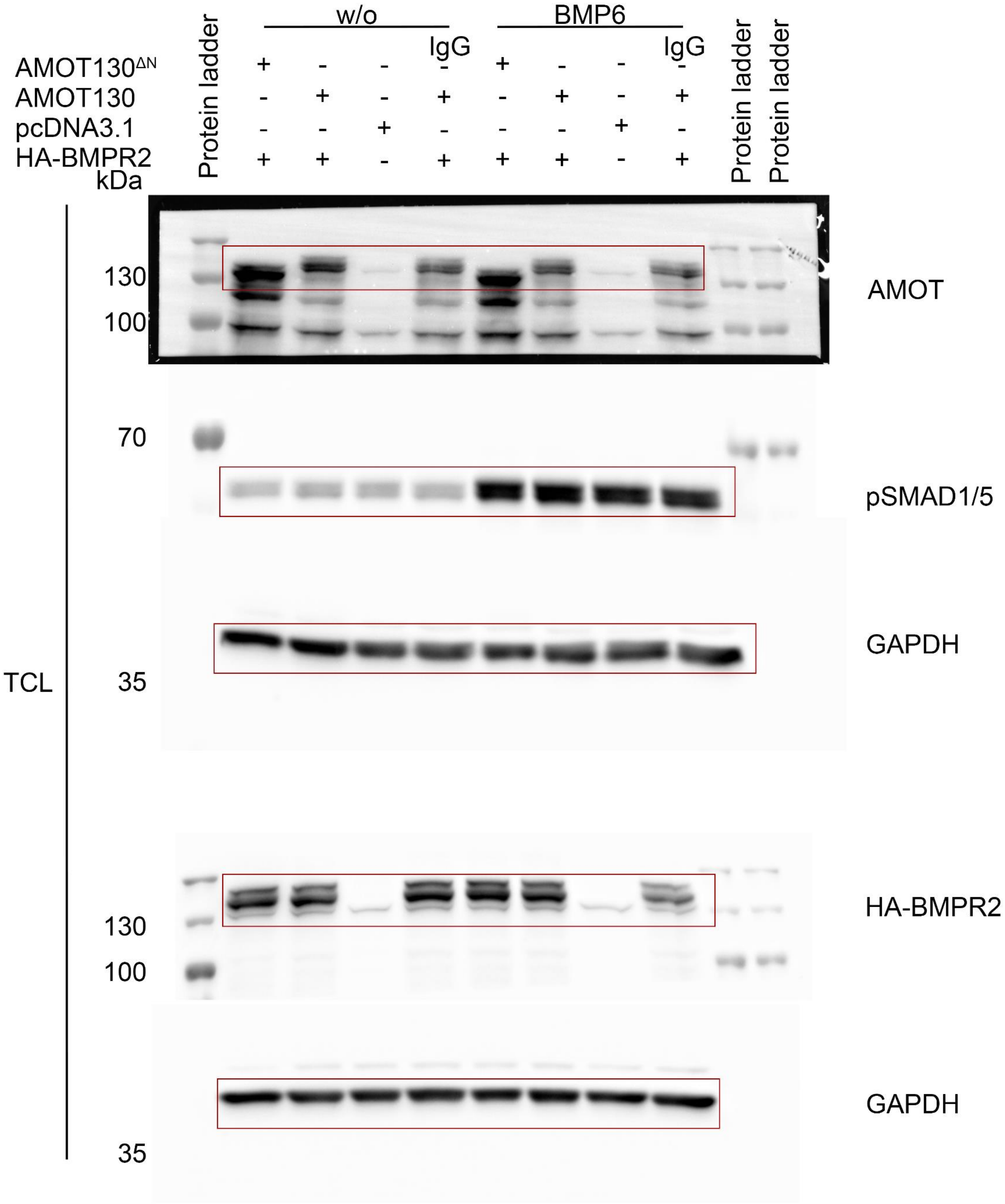

Full unedited blot for Figure 5 A_PD

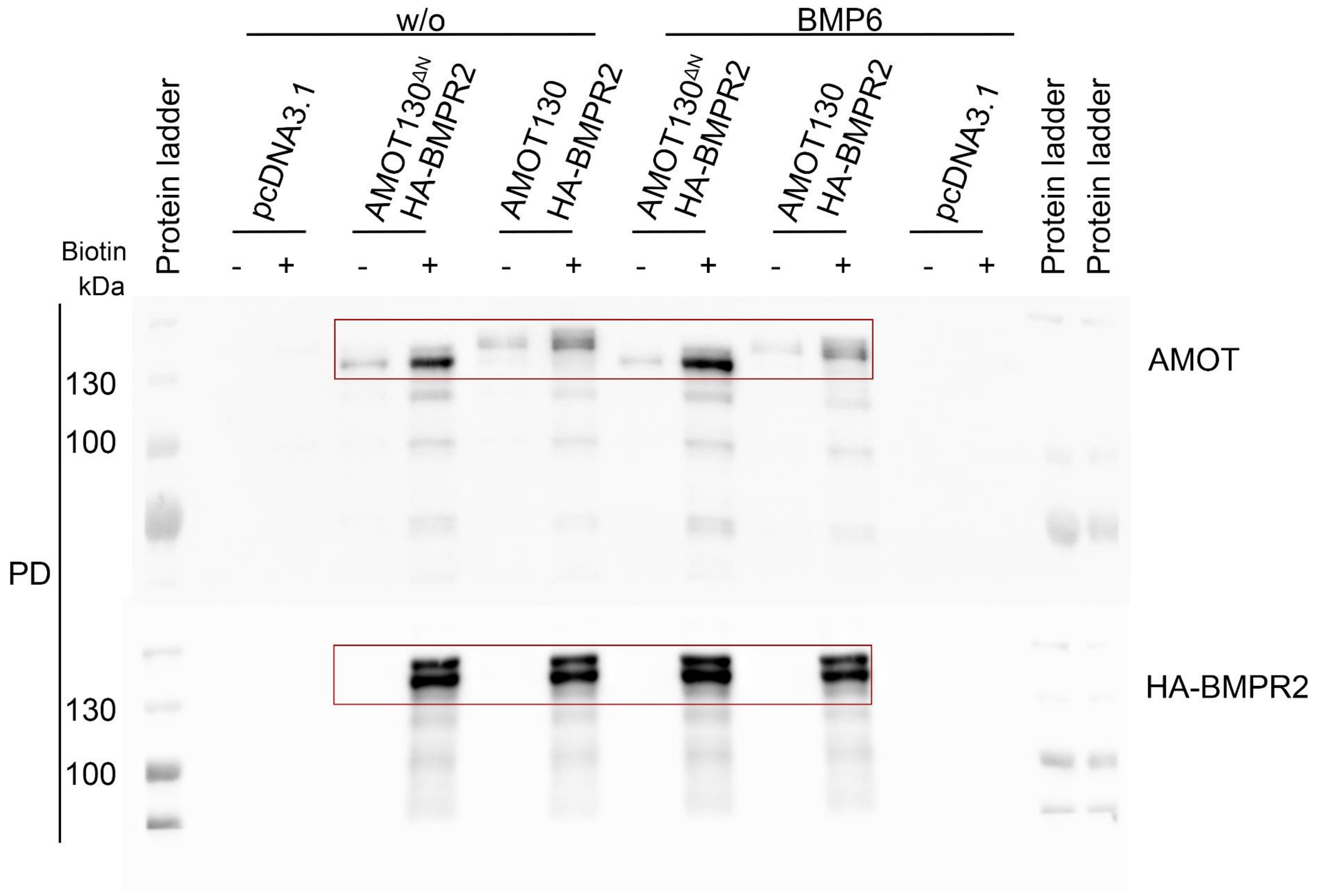

Full unedited blot for Figure 5 A_TCL

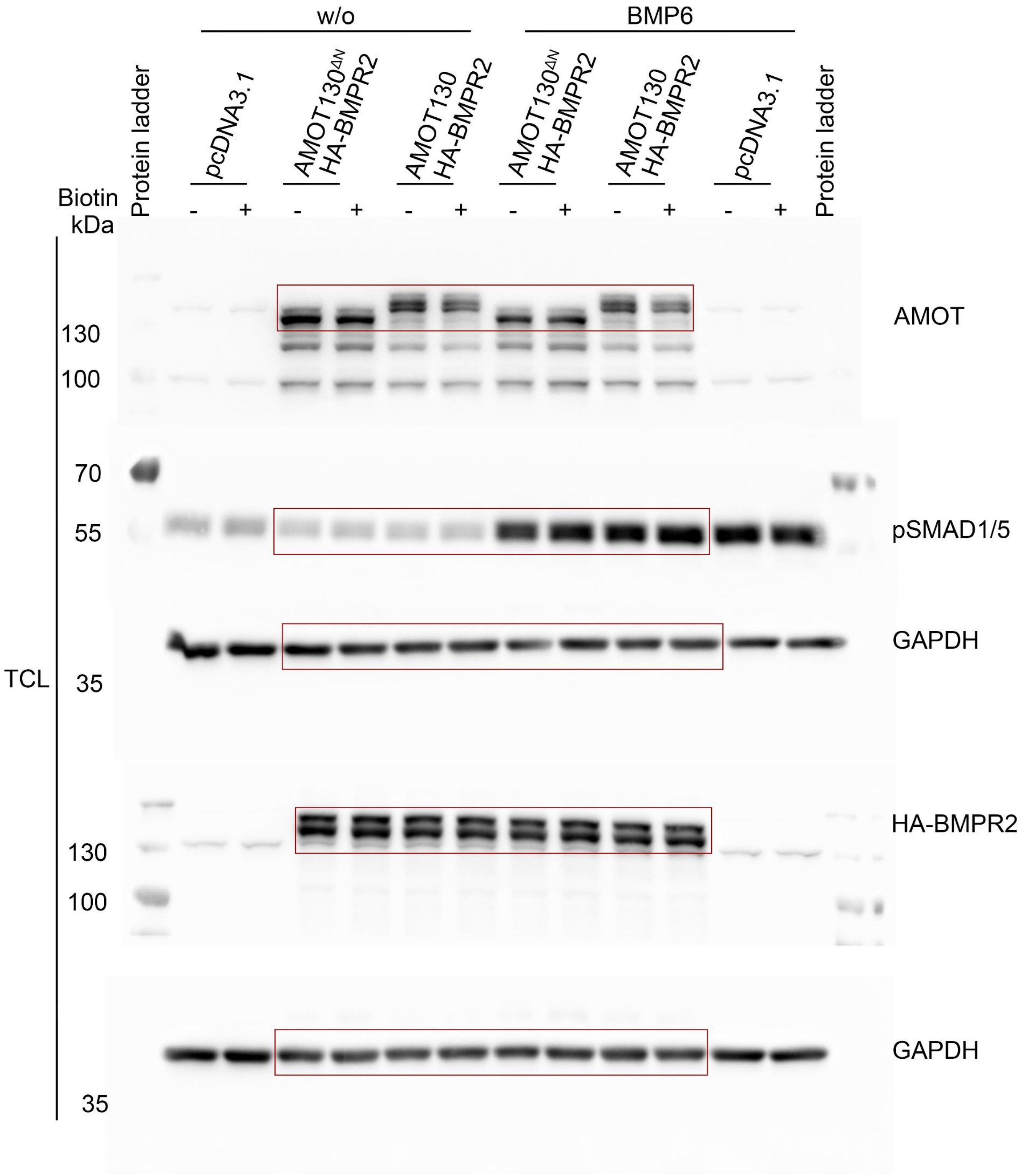

Full unedited blot for Supplemental Figure 3 A

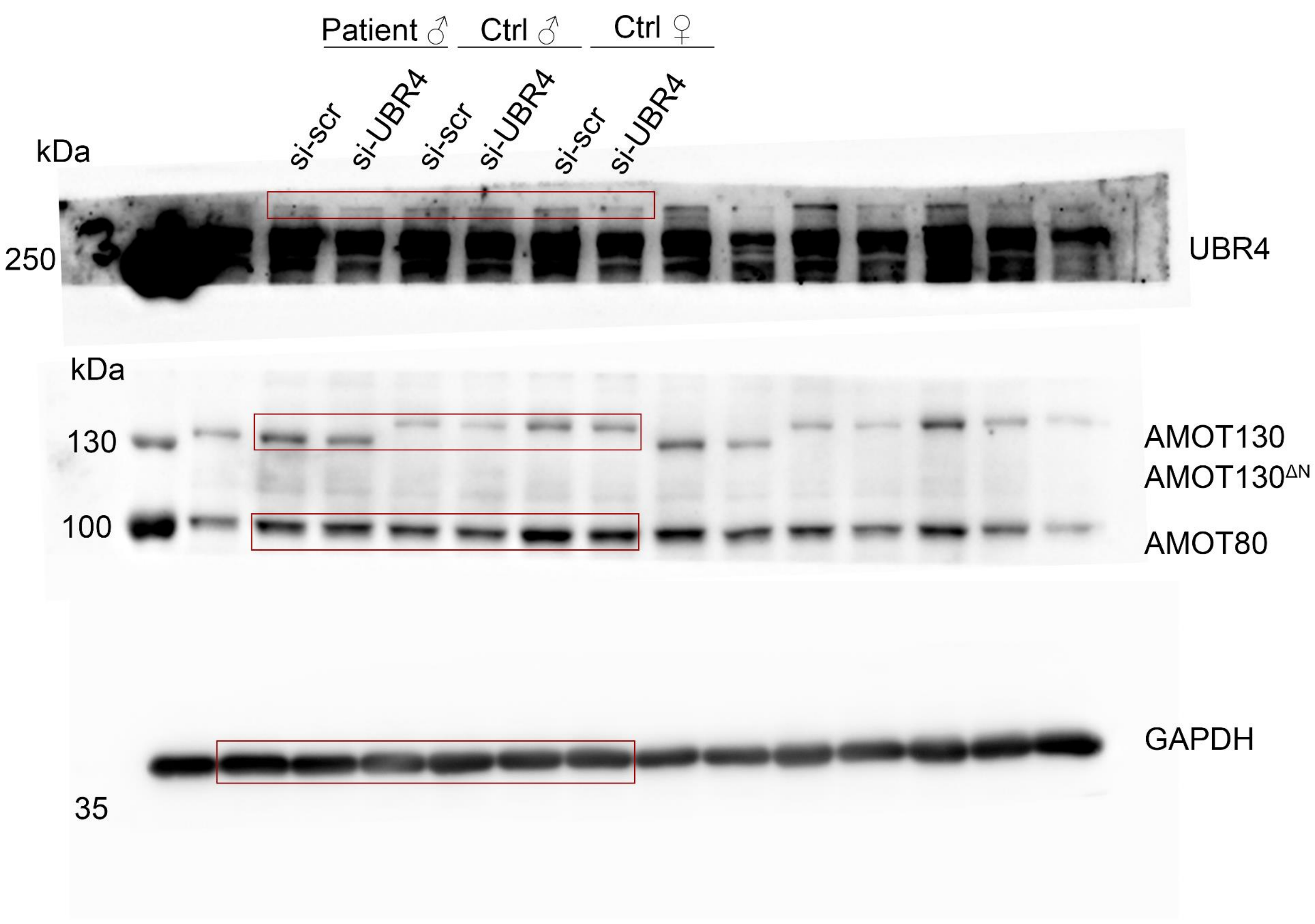

Full unedited blot for Supplemental Figure 4 A

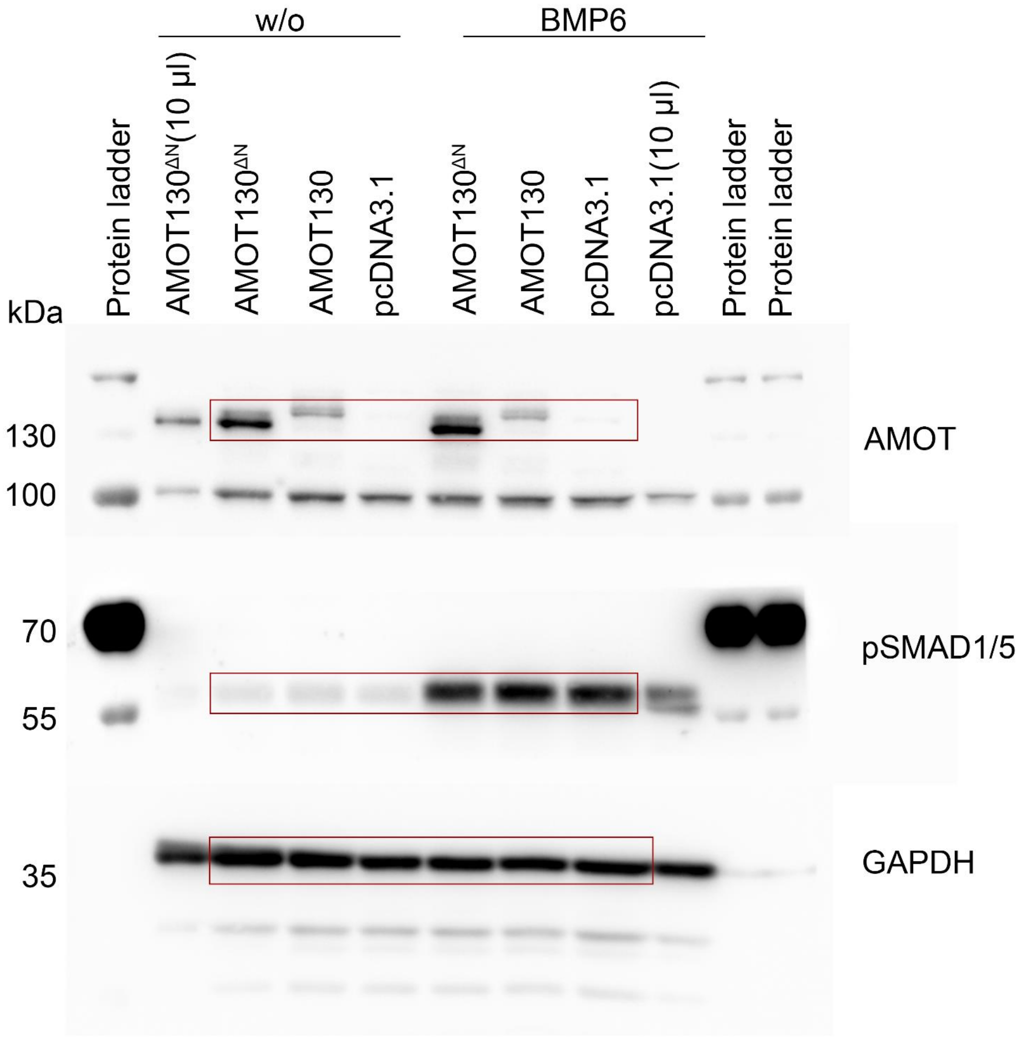

Full unedited blot for Supplemental Figure 4 C

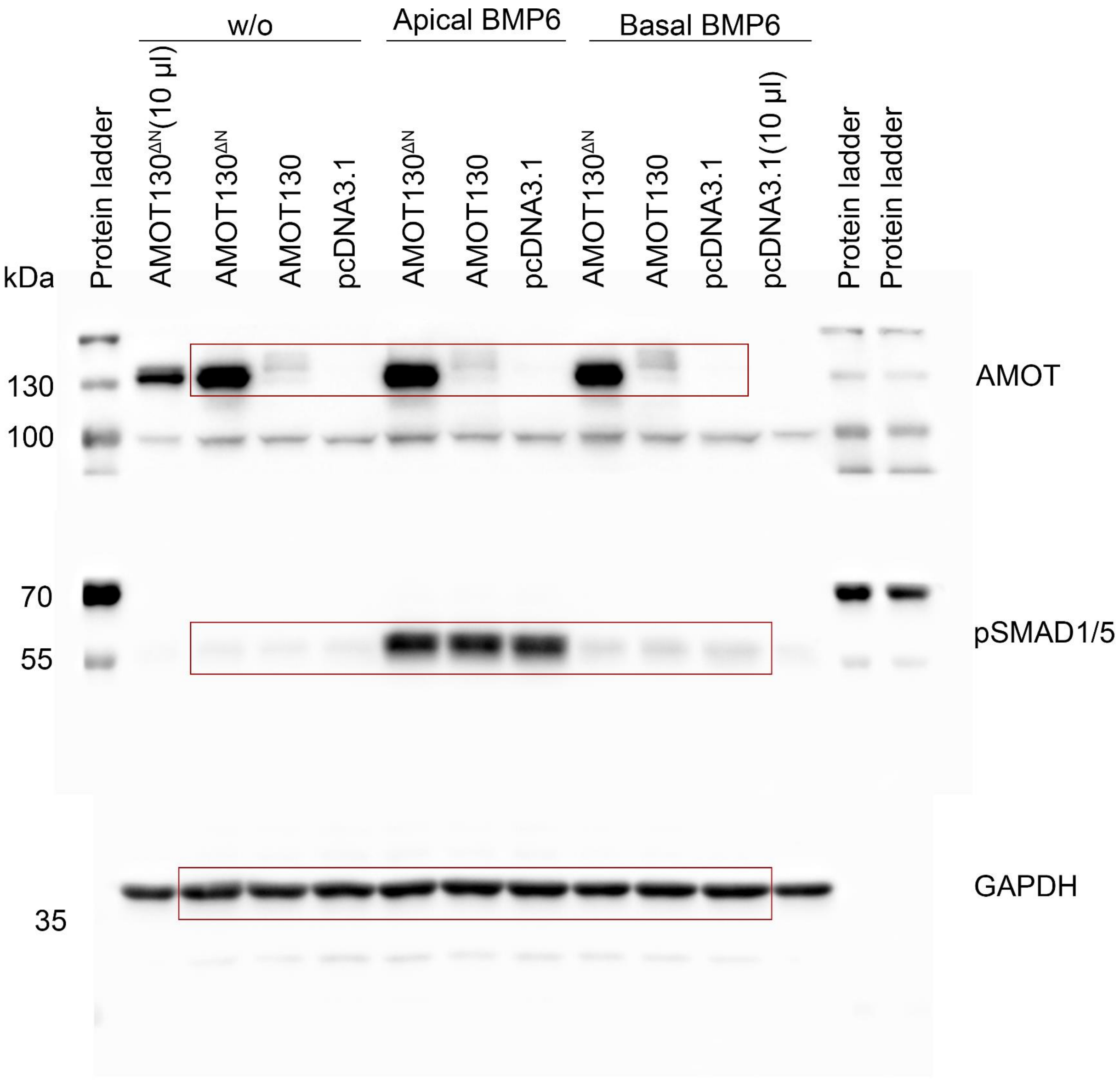

Full unedited blot for Supplemental Figure 5 D

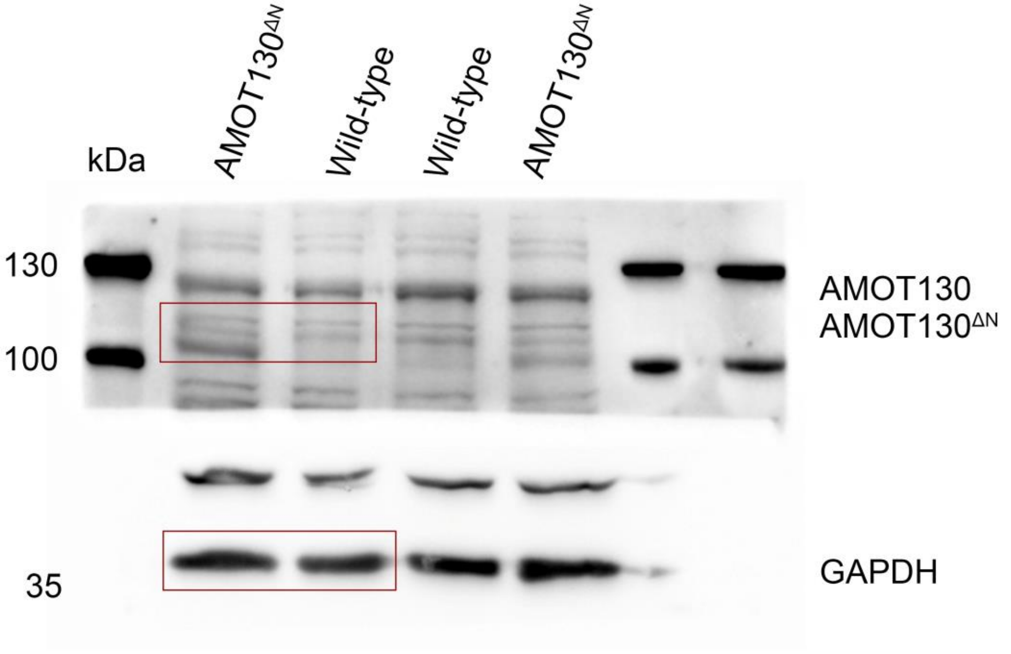

Full unedited blot for Supplemental Figure 5 E

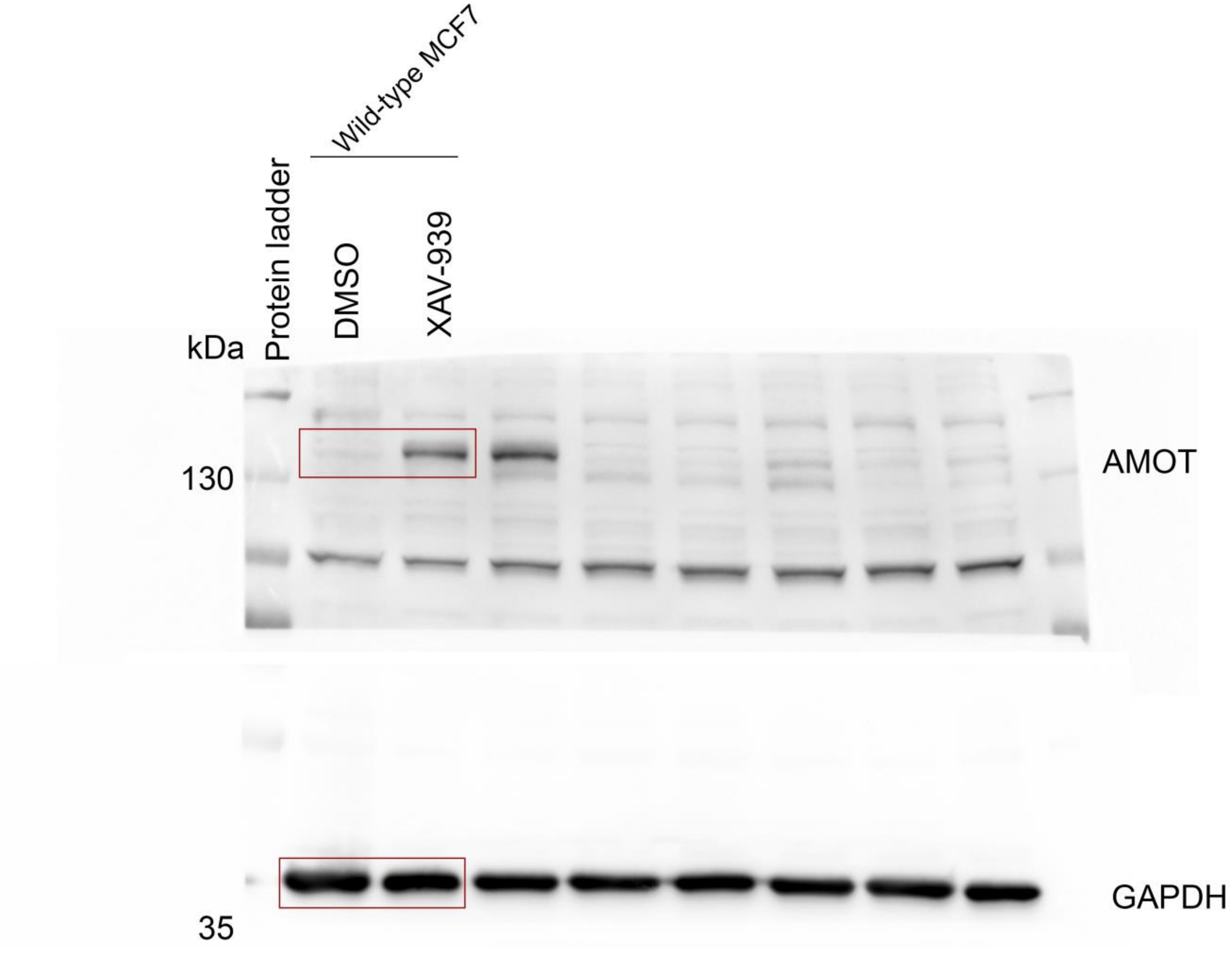

Full unedited blot for Supplemental Figure 5 H

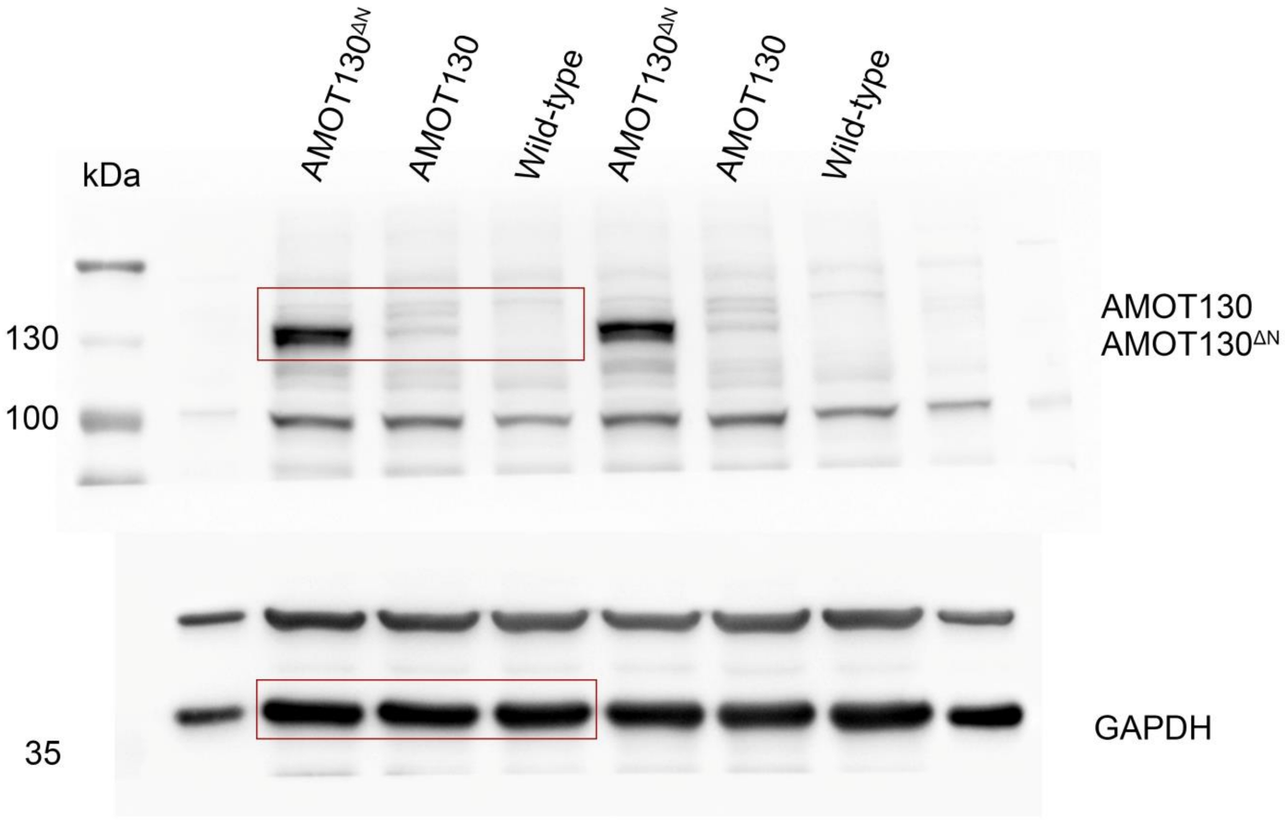

Supplement: Unedited blot and gel images [file jci-135-179438-s068.pdf]
